# YIF1A activates mTORC1 signaling to promote cellular senescence

**DOI:** 10.1038/s41419-026-09034-z

**Published:** 2026-06-23

**Authors:** Xiaogang Zhang, Luying Liu, Mengdi Shang, Bin Hu, Shu Zhu, Xi Wang, Jieying Liu, Yanchun Han, Xiaodan Wei, Qi Cao, Fan Li, Lijie Gao, Jingyu Sun, Jiaqi Yu, Chentai Tan, Menghua Dong, Tie-Shan Tang, Jiu-Qiang Wang

**Affiliations:** 1 School of Special Education and Rehabilitation, Shandong Medical and Pharmaceutical University, Yantai, China; 2 School of Life Sciences and Health, University of Health and Rehabilitation Sciences, Qingdao, China; 3 Peninsular Cancer Center, Shandong Medical and Pharmaceutical University, Yantai, China; 4 The First School of Clinical Medicine, Shandong Medical and Pharmaceutical University, Binzhou, China; 5 Institute of Science and Technology Planning and Foresight, Chinese Academy of Science and Technology for Development, Beijing, China; 6 School of Basic Medical, Shandong Medical and Pharmaceutical University, Yantai, China; 7 School of Nursing, Zibo Polytechnic University, Zibo, China; 8 Key Laboratory of Organ Regeneration and Reconstruction, Institute of Zoology, Chinese Academy of Sciences, Beijing, China; 9 University of Chinese Academy of Sciences, Beijing, China; 10 The Second Medical College, Shandong Medical and Pharmaceutical University, Yantai, China; 11 Beijing Institute for Stem Cell and Regenerative Medicine, Beijing, China; 12 Shandong Key Lab of Complex Medical Intelligence and Aging, Yantai, China

**Keywords:** Senescence, Golgi

## Abstract

The mechanistic target of rapamycin complex 1 (mTORC1) serves as a central metabolic hub that integrates nutrient signals and orchestrates cellular metabolism to regulate many fundamental cell processes. While mTORC1 activation is known to occur both on lysosomal membranes and at the Golgi apparatus in response to environmental cues, the molecular mechanisms governing its Golgi-associated activation remain poorly understood. In this study, we identified YIF1A as a novel Golgi-localized regulator of growth factor-mediated mTORC1 signaling. Mechanistically, YIF1A interacted with the E3 ubiquitin ligase RNF126 to facilitate K48-linked polyubiquitination of G3BP1/2, thereby promoting mTORC1 activation. Genetic depletion of either YIF1A or RNF126 stabilized G3BP1/2 proteins and significantly impaired mTORC1 activity. Notably, *YIF1A* knockdown conferred resistance to etoposide- and doxorubicin-induced cellular senescence. The evolutionary conservation of this pathway was demonstrated by extended or shortened lifespan in *Caenorhabditis elegans* lacking or overexpressing *yif-1*, the invertebrate ortholog of YIF1A. Our findings not only elucidate a previously unrecognized Golgi-specific regulatory axis for mTORC1 activation but also suggest YIF1A as a potential therapeutic target for modulating aging-related pathologies.

## Introduction

The mechanistic target of rapamycin complex 1 (mTORC1) is a master growth regulator that can respond to various environmental cues, including nutrients and growth factors. Growth factors can activate the mTORC1 signaling pathway via the phosphatidylinositol-3-kinase (PI3K)-protein kinase B (PKB/AKT) signaling pathway. Following phosphorylation at Ser473 [1, 2], the activated AKT1 phosphorylates tuberous sclerosis complex 2 (TSC2), thus blocking its association with TSC1 [3–5]. The TSC1/2 can form a heterotrimeric complex with TBC1 domain family member 7 (TBC1D7) [6–9], which is tethered to the lysosome by G3BP1/2 [10]. The TSC complex functions as a GTPase-activating protein (GAP) towards the small GTPase Rheb (Ras homolog-mTORC1 binding) [11], which is required for mTORC1 activation [12]. After being activated, mTORC1 directly phosphorylates its substrates, such as S6K1, unc-51 like autophagy activating kinase 1 (ULK1), and transcription factor EB (TFEB), to regulate cell growth and metabolism, such as protein synthesis, lipid metabolism, nucleotide metabolism, and glucose metabolism [13].

The Golgi apparatus, a central sorting station for newly synthesized membrane and secretory proteins, has emerged as a lysosome-independent regulator of mTORC1 activity [14, 15]. In addition to responding to amino acid changes to regulate mTORC1 activity [16, 17], the Golgi apparatus may also respond to growth factors, as indicated by the localization of G3BP1/2 and TSC1/2 on this organelle [10]. This spatial organization raises the intriguing possibility of Golgi-mediated integration of nutrient and growth factor signals. Nevertheless, the precise molecular mechanisms underlying mTORC1 regulation at the Golgi remain to be fully elucidated.

RNF126, an E3 ubiquitin ligase with established roles in cytoplasmic protein quality control and endosomal sorting [18, 19], exhibits emerging functional connections to Golgi apparatus-associated pathways. BAG6 (BAT3/Scythe), a key molecular partner of RNF126, has been demonstrated to be crucial for maintaining the structural and functional integrity of the Golgi apparatus complex [20]. Additionally, RAB8, another RNF126-interacting protein, serves as a critical regulator of vesicular trafficking, facilitating the transport of cargo proteins from the trans-Golgi network to their destination membranes [21]. Strikingly, despite these associations, RNF126 depletion induces only subtle Golgi structural perturbations [18], suggesting that its primary role is not in organelle maintenance but in regulating dynamic signaling circuits.

Cellular senescence is an evolutionarily conserved process, which is characterized by irreversible cell cycle arrest, enhanced secretion of senescence-associated secretory phenotype (SASP) factors, and progressive cellular morphological changes [22]. Accumulating evidence has established the vital role of mTORC1 in cellular senescence. In senescent cells, mTORC1 activity is persistently activated [23], subsequently leading to cell cycle arrest by promoting the translation of p21 (a cyclin-dependent kinase (CDK) inhibitor), an increase in p53 protein levels [24], by increasing transcriptional DNA damage [25], or by up-regulating p16 ^INK4A^ (a cyclin-dependent kinase (CDK) inhibitor) [26]. Additionally, an abnormal Golgi apparatus has been implicated in cellular senescence. Cellular senescence results in an expanded morphology of the Golgi apparatus [27]. Moreover, abnormal structure and vesicular function of the Golgi apparatus contribute to cellular senescence [28]. Elevated expressions of many Golgi apparatus-localized proteins, such as golgins A1 and A4 (GOLGA1/4), glutamate-receptor-interacting protein (GRIP), and coiled-coil-domain-containing 1 and 2 (GCC1/2), are also observed in senescent cells [27]. Given the localization of mTORC1 at the Golgi apparatus, it remains unknown whether any Golgi-resident proteins regulate aging through mTORC1

YIF1A, a member of the Yip1 domain family (YIPF) of proteins, exhibits dual localization at both the Golgi apparatus and the endoplasmic reticulum-Golgi intermediate compartments (ERGICs) [29–31]. Emerging evidence demonstrates that YIF1A expression is markedly upregulated in cellular senescence models and aging mouse tissues [32–36]. Additionally, significant up-regulation of YIF1A is also observed in various cancer types [37–39]. Given the established role of the mTORC1 signaling pathway in modulating cellular senescence and cancer progression [13, 40–43], we sought to investigate the potential functional interplay between YIF1A and mTORC1 signaling.

In this study, we established YIF1A as a novel positive modulator of mTORC1 signaling. Mechanistically, YIF1A facilitates K48-linked ubiquitination of G3BP1/2 via interaction with the E3 ubiquitin ligase RNF126. Additionally, our findings highlight the critical role of the YIF1A-mediated mTORC1 signaling pathway in regulating cellular aging processes. These findings underscore the therapeutic potential of targeting the YIF1A-mTORC1 signaling nexus to develop interventions against age-related pathologies.

## Results

### YIF1A positively regulates growth factor-induced mTORC1 signaling

**Fig. 1:**
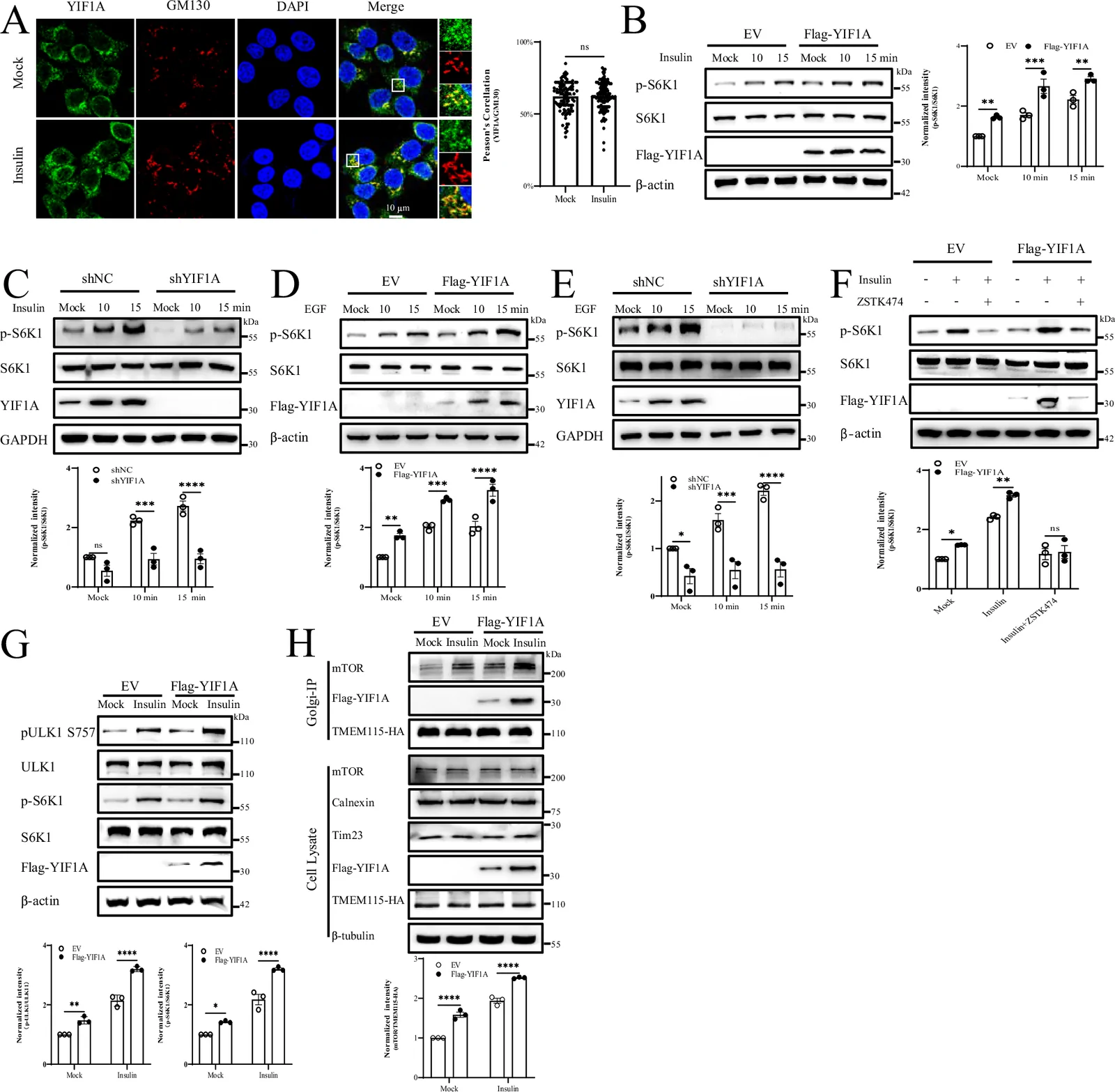
YIF1A positively regulates growth factor-mediated mTORC1 signal pathway. **A** MCF7 cells were starved of serum for 50 min, or starved and re-stimulated with 100 nM insulin for 15 min. The cells were then fixed and immunostained with the related antibodies. The co-localization between YIF1A and GM130 was quantified using Pearson’s correlation. Each dot is represented by an individual data. **B** MCF7 cells expressing empty vector (EV) or Flag-YIF1A were starved of serum for 50 min, or starved and re-stimulated with 100 nM insulin for 10 or 15 min (*n* = 3 of independent experiments). **C** MCF7 expressing negative control (shNC) or shYIF1A were treated as described in Fig. (**B**) (*n* = 3 of independent experiments). **D** MCF7 cells expressing EV or Flag-YIF1A were starved of serum for 50 min, or starved and re-stimulated with 20 nM EGF for 10 or 15 min (*n* = 3 of independent experiments). **E** MCF7 cells expressing shNC or shYIF1A were treated as described in Fig. (**D**) (*n* = 3 of independent experiments). **F** MCF7 cells expressing EV or Flag-YIF1A were starved of serum for 50 min, or starved and re-stimulated with 100 nM insulin for 15 min. DMSO or ZSTK474 (100 nM) was pre-added for 50 min before addition of insulin (*n* = 3 of independent experiments). **G** MCF7 cells expressing EV or Flag-YIF1A were starved of serum for 50 min, or starved and re-stimulated with 100 nM insulin for 15 min (*n* = 3 of independent experiments). **H** HEK293T cells stably expressing TMEM115-HA were transfected with either EV or Flag-YIF1A plasmids. Following transfection, the cells were subjected to serum starvation for 50 min, with or without subsequent stimulation using 100 nM insulin for 15 min. The cells were then homogenized to isolate Golgi apparatus by immunoprecipitation using HA beads (*n* = 3 of independent experiments). The data were expressed as mean ± SE. ns: not significant; *P ≤ 0.05; **P ≤ 0.01; *** *P* ≤ 0.001; **** *P* < 0.0001.

To explore the role of YIF1A in the mTORC1 signaling pathway, we first examined its localization in response to insulin stimulation. Consistent with the previous reports [29–31], YIF1A primarily localized at the Golgi apparatus and ERGIC (Figs. 1A and S1A), which were not affected upon insulin stimulation (Figs. 1A and S1A). Additionally, YIF1A exhibited minimal colocalization with Lamp1 (lysosome marker) (Fig. S1B). As expected [7, 44], insulin stimulation could promote the phosphorylation of S6K1 at T389 (p-S6K1), a direct downstream substrate of mTORC1 (Fig. 1B). Overexpression of YIF1A greatly enhanced insulin-stimulated p-S6K1 in MCF7 cells compared to control groups (Fig. 1B), and YIF1A protein levels also increased upon insulin stimulation (Fig. 1B). However, insulin stimulation has little effect on YIF1A transcription (Fig. S1C). Similar enhancement was also observed in Sum159PT, MDA-MB-231, HeLa and NCI-H1299 cells (Fig. S2A–D). Conversely, the knockdown of *YIF1A* suppressed p-S6K1 in the detected cells (Figs. 1C and S2E, F). Re-expression of shRNA-resistant *YIF1A* restored the decreased pS6K1 in YIF1A-silenced cells (Fig. S2G). Additionally, YIF1A-mediated mTORC1 activation also occurred in response to epidermal growth factor (EGF) stimulation (Fig. 1D, E). These results indicated that the YIF1A positively regulated the mTORC1 signaling pathway. Consistent with this mechanism, rapamycin, the inhibitor of mTOR, attenuated YIF1A-induced hyperphosphorylation of S6K1 at T389 (Fig. S2H), indicating that YIF1A functioned upstream of mTORC1. To determine whether YIF1A-mediated mTORC1 signaling was regulated by PI3K, we treated cells with ZSTK474, an ATP-competitive inhibitor of PI3K and a weak mTOR inhibitor [45]. ZSTK474 treatment significantly attenuated YIF1A overexpression-induced mTORC1 activation (Fig. 1F). Additionally, YIF1A overexpression also enhanced the phosphorylation of ULK1 at S757 (Fig. 1G), another downstream substrate of mTORC1. To further explore the role of YIF1A in mTORC1 signaling, Golgi apparatus was purified from YIF1A-overexpressed cells using Golgi-IP [46]. The results showed that insulin treatment elevated YIF1A protein levels at the Golgi apparatus, coinciding with a significant increase in Golgi-localized mTOR (Fig. S2I), which was further confirmed in YIF1A-overexpressing cells (Fig. 1H). Brefeldin A (BFA), an inhibitor of endoplasmic reticulum (ER)-to-Golgi transport that causes Golgi protein redistribution to the ER [47], diminished the enhanced mTORC1 activity resulting from YIF1A overexpression (Fig. S2J), highlighting the importance of YIF1A localization at Golgi apparatus for mTORC1 activity.

### YIF1A facilitates the degradation of G3BP1/2

**Fig. 2:**
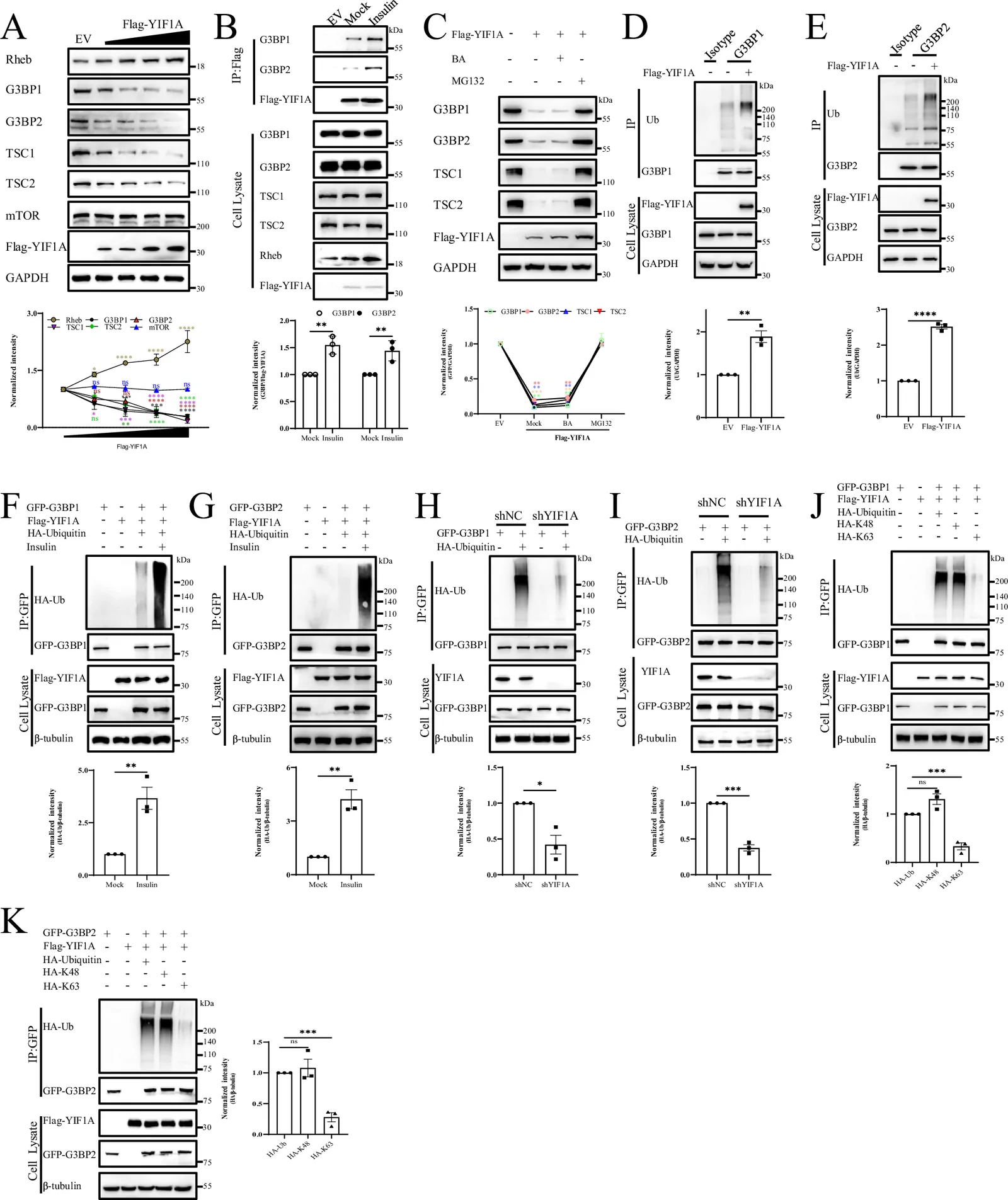
YIF1A promotes the K48-linked ubiquitination of G3BP1/2. **A** Sum159PT cells were transfected with the increased doses of Flag-YIF1A for 24 h prior to being lysed. **B** HEK293T cells overexpressing with or without Flag-YIF1A were starved of serum for 15 h, or starved and re-stimulated with 100 nM insulin for 15 min. The collected cells were lysed and added Flag M2 beads to perform immunoprecipitation (IP). MG132 (10 μM) was added to cell cultures for 15 h before addition of 100 nM insulin. **C** Sum159PT cells expressing EV or Flag-YIF1A were incubated with DMSO, 1 μM Bafilomycin A1 (BA) or 10 μM MG132 for 15 h prior to being lysed. HEK293T cells expressing EV or Flag-YIF1A were lysed to perform denaturing IP using antibody against G3BP1 (**D**) or G3BP2 (**E**), followed by purification using Protein A/G beads. MG132 (10 μM) was added to cell cultures for 15 h prior to cell lysis. F-G, HEK293T cells expressing GFP-G3BP1 (**F**) or GFP-G3BP2 (**G**), along with or without Flag-YIF1A and HA-ubiquitin, were serum-starved for 15 h, or starved and re-stimulated with 100 nM insulin for 15 min. Cells were collected and lysed to perform denaturing IP using GFP beads. MG132 (10 μM) was added to the culture medium for 15 h prior to cell lysis. The shNC or shYIF1A HEK293T cells expressing GFP-G3BP1(**H**) or GFP-G3BP2 (**I**) with or without HA-ubiquitin were collected to perform denaturing IP using GFP beads. MG132 (10 μM) was added to the culture medium for 15 h prior to cell lysis. **J, K** HEK293T cells expressing the indicated proteins were treated as described in Fig. (**H**). The data were obtained from three independent experiments and expressed as mean ± SE. ns: not significant; *P ≤ 0.05; **P ≤ 0.01; ***P ≤ 0.001; ****P < 0.0001.

Given the role of Rheb, G3BP1/2 and TSC1/2 in growth factor-mediated mTORC1 signaling pathway, we investigated the impact of YIF1A on their protein levels. Interestingly, YIF1A overexpression reduced the protein levels of G3BP1/2 and TSC1/2 in a dose-dependent manner while concurrently increasing Rheb levels (Fig. 2A). Conversely, *YIF1A* knockdown led to opposite results (Fig. S3A). However, YIF1A silencing minimally affected their transcript levels (Fig. S3B). Immunoprecipitation showed that YIF1A could interact with endogenous or exogenous G3BP1/2 (Figs. 2B and S3C). These findings indicated that YIF1A regulated mTORC1 signaling pathway by targeting G3BP1/2.

We next aimed to investigate how YIF1A affected the protein levels of G3BP1/2. G3BP1/2 could localized at Golgi apparatus (Fig. S3D, E), and their localization at the Golgi apparatus was unaffected by YIF1A silencing (Fig. S3F, G). The YIF1A-mediated decrease in G3BP1/2 protein levels was blocked by the ubiquitin-proteasome inhibitor MG132, but not by the lysosomal inhibitor bafilomycin A1 (BA) (Figs. 2C and S3H–J), indicating that YIF1A promoted ubiquitin-proteasome-mediated degradation of G3BP1/2. Additionally, MG132 also reversed the reduced TSC1/2 levels in YIF1A-overexpressing cells (Fig. 2C), suggesting the involvement of ubiquitin-proteasome in TSC1/2 degradation.

### YIF1A promotes K48-linked ubiquitination of G3BP1/2

Since G3BP1/2 underwent ubiquitin-proteasome-mediated degradation upon YIF1A overexpression, we then wanted to know whether they underwent ubiquitination within the insulin-stimulated mTORC1 signaling pathway. Given that HEK293T cells have been widely used to study protein ubiquitination [48–52], we employed this system to detect the ubiquitination of G3BP1/2. Under serum-free conditions, G3BP1/2 underwent ubiquitination, which was enhanced upon insulin stimulation (Fig. S3K, L). As expected, YIF1A overexpression significantly increased G3BP1/2 ubiquitination (Fig. 2F, G), and this effect was further augmented by insulin stimulation (Fig. 2F, G). Conversely, YIF1A silencing markedly reduced G3BP1/2 ubiquitination levels (Fig. 2H, I). To further validate these results, we also detected their endogenous ubiquitination. The results showed that insulin stimulation indeed promoted the ubiquitination of G3BP1/2, which was attenuated upon YIF1A deficiency (Fig. S3M, N). Subsequently, we applied K48 and K63 ubiquitin, which indicate that the 48th or 63rd amino acid of wild-type ubiquitin is K and the other lysines were mutated to arginines [53], to determine the ubiquitination type of G3BP1/2. The results showed that YIF1A overexpression primarily promoted the K48-linked ubiquitination of G3BP1/2 (Fig. 2J, K).

### YIF1A interacts with RNF126

**Fig. 3:**
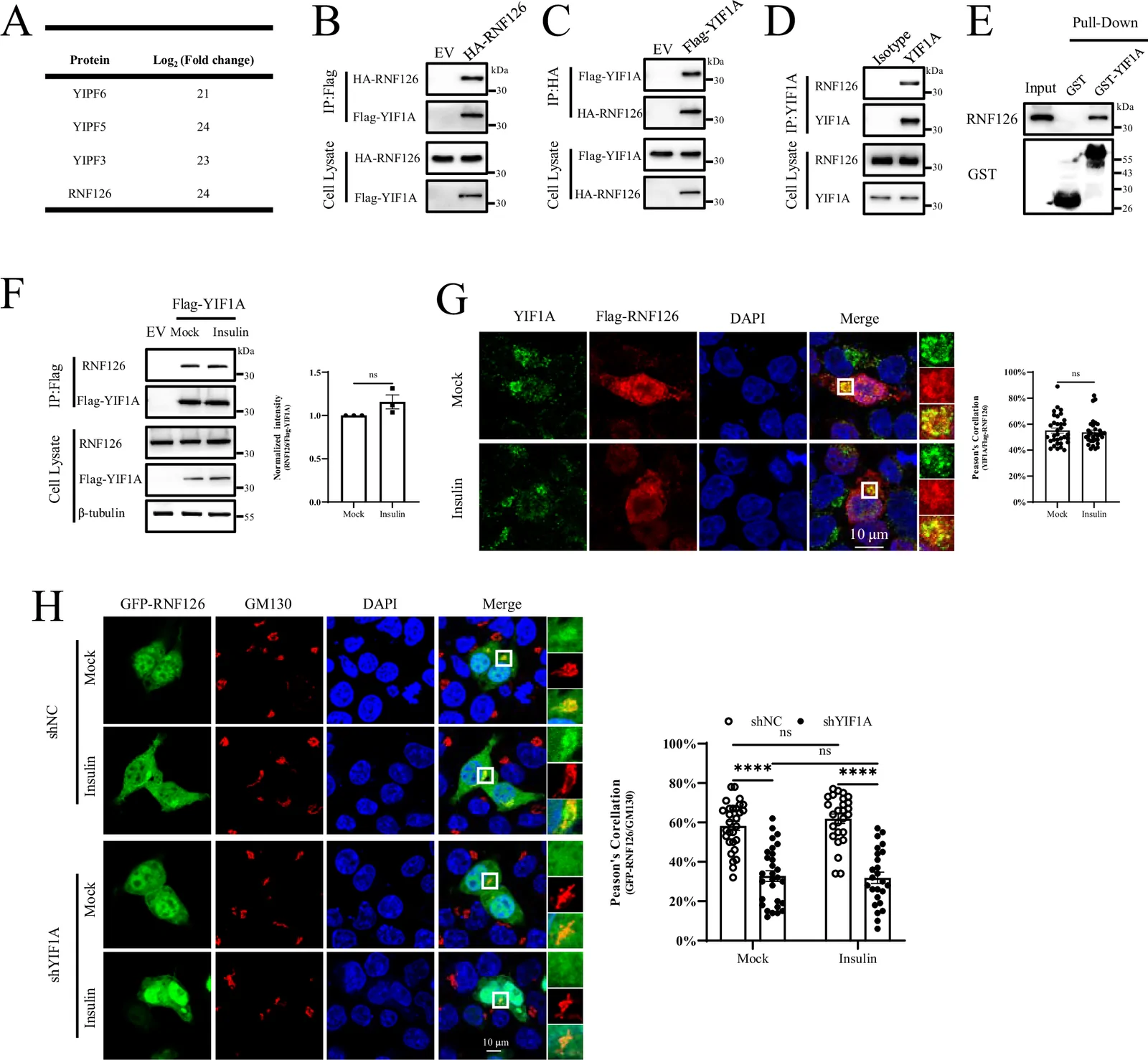
YIF1A interacts withRNF126. **A** Identification of YIF1A-associated proteins via mass spectrometry. **B** HEK293T cells expressing Flag-YIF1A with EV or HA-RNF126 were collected and lysed to perform immunoprecipitation (IP) using M2 flag beads (*n* = 3 of independent experiments). **C** HEK293T cells expressing HA-RNF126 with EV or Flag-YIF1A were collected and lysed to perform IP using HA beads (*n* = 3 of independent experiments). **D** The cell lysates from HEK293T cells were incubated with or without anti-YIF1A antibodies, followed by immunoprecipitation using Protein A/G magnetic beads (*n* = 3 of independent experiments). **E** The purified GST or GST-YIF1A proteins were incubated with cell lysate from HEK293T cells to perform GST Pull-down using GST beads (*n* = 3 of independent experiments). **F** HEK293T cells expressing EV or Flag-YIF1A were starved for 15 h, or starved or re-stimulated with 100 nM insulin for 15 min. The cells were collected and lysed to perform IP using M2 flag beads (*n* = 3 of independent experiments). **G** HEK293T cells expressing Flag-RNF126 were starved for 15 h, or starved or re-stimulated with 100 nM insulin for 15 min. The cells were then fixed to perform immunofluorescence. Each dot is represented by an individual data. **H** The shNC and shYIF1A HEK293T cells expressing GFP-RNF126 were treated as described in Fig. (**G**). Each dot is represented by an individual data. The data were expressed as mean ± SE. ns: not significant; ****P < 0.0001.

To screen for YIF1A-associated E3 ubiquitin ligases, we performed immunoprecipitation of YIF1A from HEK293T cells followed by mass spectrometry. This analysis not only confirmed established YIF1A interactors, such as YIPF3, YIPF4, and YIPF6 [29, 30], but also identified RNF126 as a newly identified binding partner (Fig. 3A). The interaction between exogenously expressed YIF1A and RNF126 was demonstrated by reciprocal co-immunoprecipitation (co-IP) assays (Fig. 3B, C). Moreover, endogenous co-IP further confirmed the interaction between the native YIF1A and RNF126 (Fig. 3D), which was independently validated by GST pull-down assay (Fig. 3E). Notably, neither their interaction (Fig. 3F) nor their subcellular co-localization (Fig. 3G) was altered by insulin stimulation. While the presence of RNF126 at the Golgi apparatus was largely unaltered by insulin (Fig. S4A), it was markedly reduced upon YIF1A silencing (Fig. 3H), suggesting that the proper subcellular distribution of RNF126 functionally depends on YIF1A.

### RNF126 positively regulates mTORC1 signaling

To assess the impact of RNF126 on mTORC1 signaling, we then evaluated its effect on mTORC1 activity. RNF126 overexpression significantly enhanced insulin-stimulated p-S6K1 (Fig. S4B–D), whereas *RNF126* knockdown suppressed it (Fig. S4E–G). Furthermore, RNF126 overexpression increased mTORC1-dependent phosphorylation of ULK1 at Ser757 (Fig. S4H). Collectively, these results establish RNF126 as a positive regulator of the mTORC1 signaling pathway.

### RNF126 promotes K48-linked ubiquitination of G3BP1/2

**Fig. 4:**
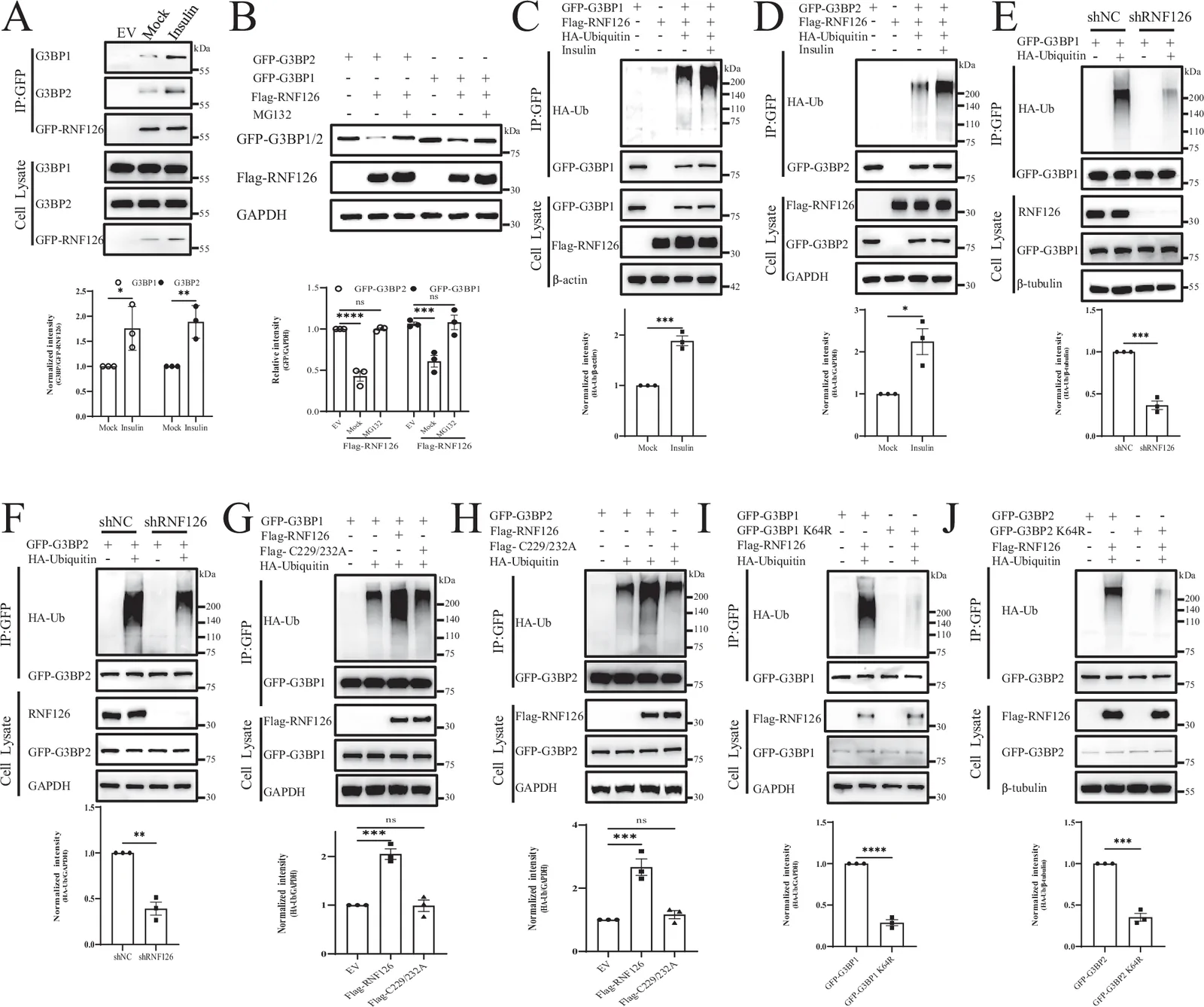
RNF126 promotes the K48-linked ubiquitination of G3BP1/2. **A** HEK293T cells expressing EV or GFP-RNF126 were starved of serum for 15 h, or starved and re-stimulated with 100 nM insulin for 15 min. The collected cells were lysed to perform IP using GFP beads.10 μM MG132 was added to medium for 15 h before addition of insulin. **B** HEK293T cells expressing GFP-G3BP1 or GFP-G3BP2 with or without Flag-RNF126 were incubated with or without 10 μM MG132 for 15 h prior to being lysed. HEK293T cells expressing GFP-G3BP1(**C**) or GFP-G3BP2 (**D**) with or without Flag-RNF126 and HA-ubiquitin were starved of serum for 15 h, and starved or re-stimulated with 100 nM insulin for 15 min. The collected cells were lysed and boiled to perform denaturing IP using GFP beads. 10 μΜ MG132 was added to the cell culture medium for 15 h prior to being lysed. The shNC or shRNF126 HEK293T cells expressing GFP-G3BP1(**E**) or GFP-G3BP2 (**F**) with or without HA-ubiquitin were collected, lysed and boiled to perform denaturing IP using GFP beads. MG132 was added to the cell culture medium for 15 h prior to being lysed. HEK293T cells expressing GFP-G3BP1 (**G**) or GFP-G3BP2 (**H**), with or without HA-ubiquitin, Flag-RNF126 or Flag-RNF126 C229/232 A (Catalytically inactive mutant) were treated as described in Fig. (**E**). **I** HEK293T cells expressing the indicated proteins were treated as described in Fig. (**E**). **J** HEK293T cells expressing the indicated proteins were treated as described in Fig. (**E**). The data were obtained from three independent experiments and expressed as mean ± SE. ns: not significant; *P ≤ 0.05; **P ≤ 0.01; ***P ≤ 0.001; ****P < 0.0001.

To determine whether RNF126 positively regulated mTORC1 signaling pathway by targeting G3BP1/2, we firstly assessed their interaction. RNF126 could interact with both endogenous and exogenous G3BP1/2 (Figs. 4A and S5A), which was enhanced upon insulin stimulation (Fig. 4A). As expected, RNF126 overexpression significantly decreased G3BP1/2 protein levels, an effect blocked by the proteasome inhibitor MG132 (Figs. 4B and S5B, C). Conversely, *RNF126* knockdown significantly increased G3BP1/2 protein levels (Fig. S5D) but had minimal effects on their mRNA levels (Fig. S5E).

Next, we examined RNF126-mediated ubiquitination of G3BP1/2. RNF126 promoted G3BP1/2 ubiquitination under serum-free conditions (Fig. 4C, D), which was further enhanced upon insulin stimulation (Fig. 4C, D). Conversely, RNF126 silencing significantly attenuated their ubiquitination (Fig. 4E, F). RNF126 primarily mediated K48-linked ubiquitination of G3BP1/2 (Fig. S5F, G). However, their ubiquitination was reduced upon overexpression of catalytically inactive mutant RNF126 (C229/232 A) (Fig. 4G, H), further confirming the catalytic role of RNF126. To identify the ubiquitinated sites on G3BP1/2, we searched the public database (www.phosphosite.org/) and identified 10 or 9 potential ubiquitinated lysine residues for G3BP1 or G3BP2 (Fig. S6A), respectively. Subsequent mutagenesis screening revealed that the K64R mutation in both G3BP1 and G3BP2 conferred resistance to RNF126-mediated degradation (Fig. S6B–E). Furthermore, the K64R mutation significantly attenuated RNF126-mediated ubiquitination of G3BP1/2 (Fig. 4I, J). Consequently, it suppressed mTORC1 activity more potently than the wild-type (WT) control (Fig. S6F, G).

### YIF1A-promoted degradation of G3BP1/2 is mediated by RNF126

**Fig. 5:**
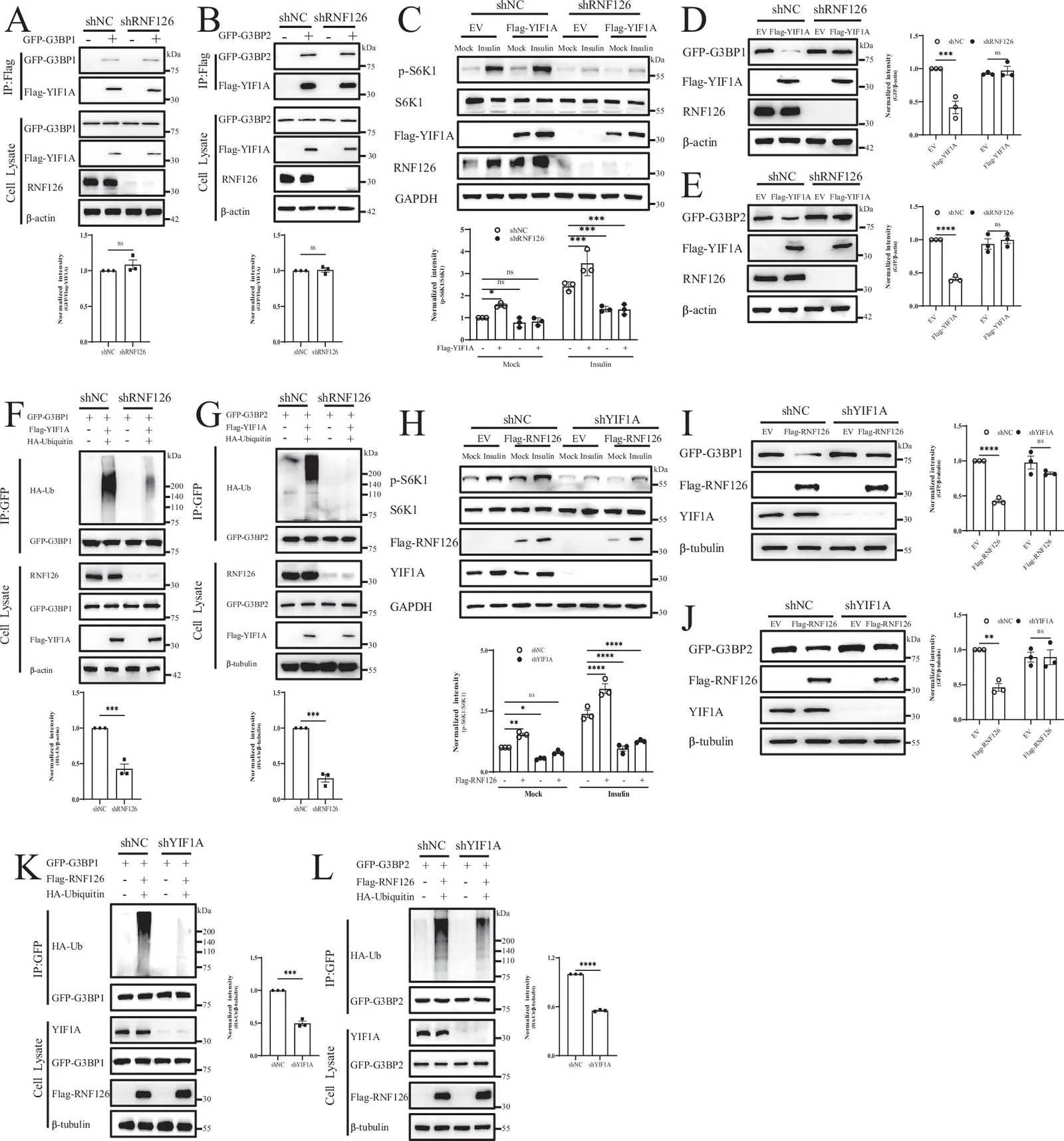
YIF1A promotes G3BP1/2 degradation in an RNF126-dependent manner. The shNC or shRNF126 HEK293T cells expressing Flag-YIF1A with or without GFP-G3BP1 (**A**) or GFP-G3BP2 (**B**) were collected and lysed to perform IP using M2 flag beads. MG132 was pre-added in cell culture for 15 h prior to being lysed. **C** The shNC and shRNF126 Sum159PT cells expressing EV or Flag-YIF1A were starved of serum for 50 min, or starved and re-stimulated with 100 nM insulin for 15 min. The shNC or shRNF126 HEK293T cells expressing GFP-G3BP1(**D**) or GFP-G3BP2 (**E**) with or without Flag-YIF1A were collected and lysed to blot the indicated proteins. The shNC or shRNF126 HEK293T cells expressing GFP-G3BP1(**F**) or GFP-G3BP2 (**G**) with or without Flag-YIF1A and HA-ubiquitin were lysed and boiled to perform denaturing IP using GFP beads. MG132 (10 μM) was pre-added to cell culture prior to being lysed. **H** The shNC and shYIF1A MCF7 cells expressing EV or Flag-RNF126 were treated as described in Fig. (**C**). The shNC or shYIF1A HEK293T cells expressing GFP-G3BP1(**I**) or GFP-G3BP2 (**J**) with or without Flag-RNF126 were treated as described in Fig. (**D**). The shNC or shYIF1A HEK293T cells expressing GFP-G3BP1(**K**) or GFP-G3BP2 (**L**) with or without Flag-RNF126 and HA-ubiquitin were treated as described in Fig. (**F**). The data were obtained from three independent experiments and expressed as mean ± SE. ns: not significant; *P ≤ 0.05; **P ≤ 0.01; ***P ≤ 0.001; ****P < 0.0001.

To examine the role of RNF126 in the interaction between YIF1A and G3BP1/2, we accessed their interaction in RNF126 silenced cells. Co-IP assays demonstrated that the silence of RNF126 minimally affected their interaction (Fig. 5A, B). However, the enhancement of p-S6K1 induced by YIF1A overexpression was attenuated upon RNF126 silence (Fig. 5C). Furthermore, RNF126 silence prevented the decrease of G3BP1/2 protein level induced by YIF1A overexpression (Fig. 5D, E). Correspondingly, the YIF1A-mediated ubiquitination of G3BP1/2 was also reduced in RNF126 silenced cells (Fig. 5F, G). Moreover, G3BP1/2 K64R mutation couldn’t be degraded and ubiquitinated by YIF1A overexpression (Fig. S7).

To further delineate the functional relationship between YIF1A and RNF126 in regulating mTORC1 activity, we expressed RNF126 in YIF1A silenced cells to observe their effect on p-S6K1. YIF1A depletion attenuated the enhancement of insulin-stimulated mTORC1 activity mediated by RNF126 overexpression (Fig. 5H). Additionally, YIF1A silencing also compromised the RNF126 overexpression-induced degradation and ubiquitination of G3BP1/2 (Fig. 5I–L).

### YIF1A enhances cellular senescence

**Fig. 6:**
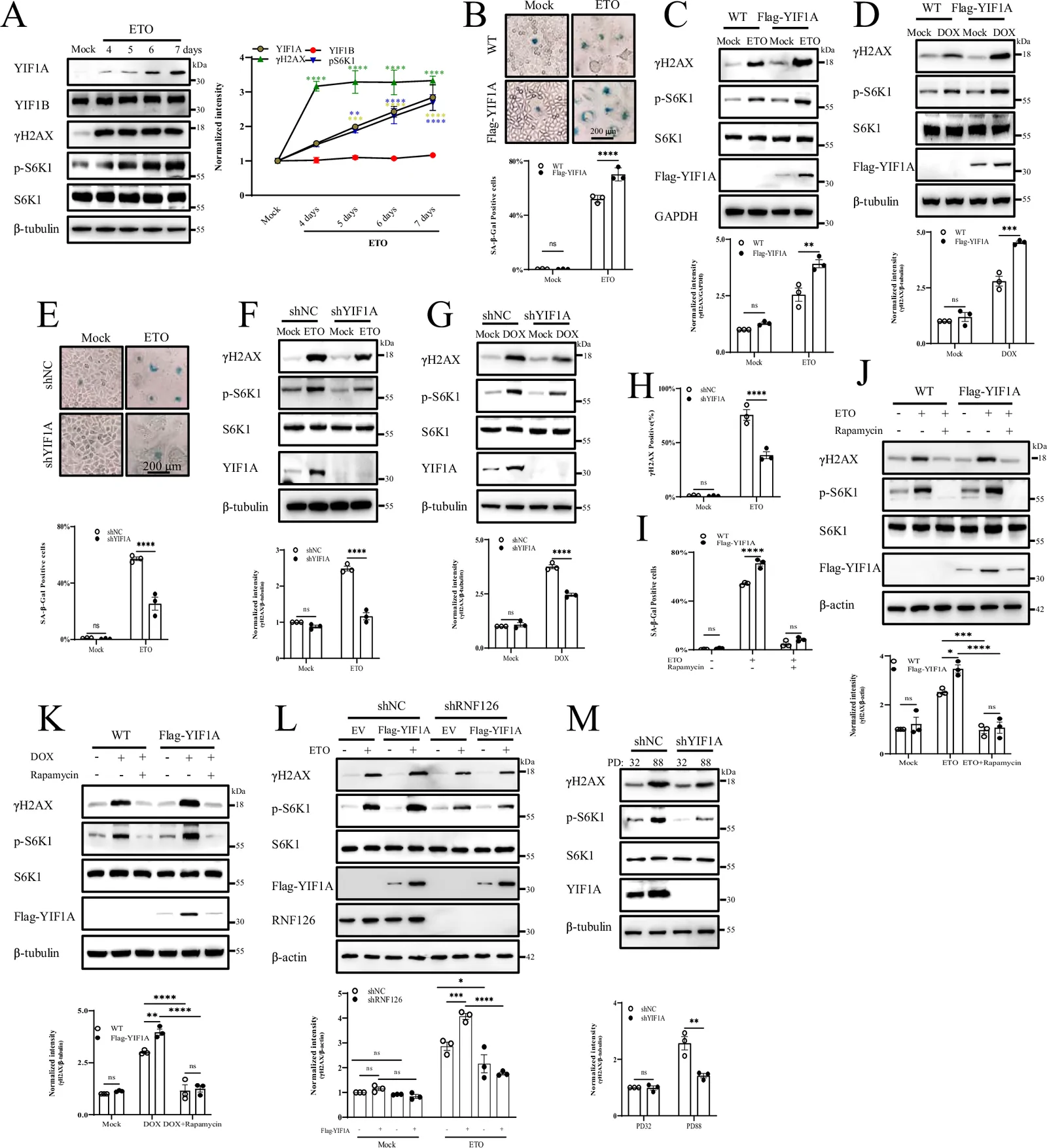
YIF1A enhances cellular senescence. **A** MCF7 cells were treated with 10 μM etoposide (ETO) for 24 h and released for 4, 5, 6 and 7 days to induce cellular senescence. **B** WT or Flag-YIF1A stably expressed MCF7 cells were treated with 2 μM ETO for 7 days, and then fixed to stain SA-β-Gal. The SA-β-Gal positive cells were counted and stasticaly analyzed using two-way ANOVA test. WT or Flag-YIF1A stably expressed MCF7 cells were treated with 2 μM ETO (**C**) or 4 μM doxorubicin (DOX) (**D**) for 7 days. **E** MCF7 cells stably expressing shNC or shYIF1A were treated with 2 μM ETO for 7 days, and then fixed to stain SA-β-Gal. MCF7 cells stably expressing shNC or shYIF1A were treated with 2 μM ETO (**F**) or 4 μM DOX (**G**) for 7 days. **H** MCF7 cells stably expressing shNC or shYIF1A were treated with 2 μM ETO for 7 days, and then fixed to perform immunofluorescence. The γH2AX positive cells were counted and statistically analyzed using two-way ANOVA test. **I** WT or Flag-YIF1A stably expressed MCF7 cells were treated with 2 μM ETO for 7 days. The cells were then fixed to stain SA-β-Gal. Rapamycin (100 nM) was added to cell culture medium following the induction of cellular senescence. WT or Flag-YIF1A stably expressed MCF7 cells were treated with 2 μM ETO (**J**) or 4 μM DOX (**K**) for 7 days. Rapamycin (100 nM) was added to cell culture medium following the induction of cellular senescence. **L** The shNC and shRNF126 MCF7 cells stably expressing EV or Flag-YIF1A were treated as in (**C**). **M** IMR90 cells at population doubling (PD) 32 were serially passaged until PD 88. The data were obtained from three independent experiments and expressed as mean ± SE. ns: not significant; *P ≤ 0.05; **P ≤ 0.01; ***P ≤ 0.001; ****P < 0.0001.

Given reports of elevated YIF1A expression in cellular senescence models and aging mouse tissues [32–36], we investigated its functional role. Consistent with prior findings [32–36], YIF1A protein levels were significantly elevated in multiple tissues (spleen, lung, liver, heart, and kidney) from aged mice (24 months) versus young mice (3 months) (Fig. S8A). In cells undergoing senescence induced by DNA-damaging agents, etoposide (ETO) or doxorubicin (DOX), YIF1A protein level markedly increased concomitantly with senescence markers γH2AX or P16 ^INK4A^ (Figs. 6A and S8B–D). Concurrently, mTORC1 activity was enchanced following senescence induction (Figs. 6A and S8B–D). Notably, YIF1B, a homolog of YIF1A, exhibited no significant changes during cellular senescence (Figs. 6A and S8B–D). YIF1A overexpression exacerbated ETO-induced senescence, evidenced by increased SA-β-galactosidase (SA-β-Gal) staining (Fig. 6B), elevated γH2AX or P16 ^INK4A^ in YIF1A-overexpressing cells (Figs. 6C, D and S8E, F). Conversely, *YIF1A* knockdown significantly reduced SA-β-Gal positivity and suppressed γH2AX and P16^INK4A^ levels (Figs. 6E, G and S8G, H), with a parallel decrease in γH2AX-positive cells in ETO-treated YIF1A-depleted cells (Figs. 6H and S8I). The pro-senescent effects of YIF1A overexpression were reversed by rapamycin (Figs. 6I–K and S8J, K) or by RNF126 knocking down (Fig. 6L). Additionally, Brefeldin A (BFA) also attenuated senescence in YIF1A-overexpressing cells (Fig. S8L). Notably, *YIF1A* knockdown also significantlty attenuated replicative senescence in IMR-90 cells (Fig. 6M).

### YIF1A has a conserved role in *C. elegans*

**Fig. 7:**
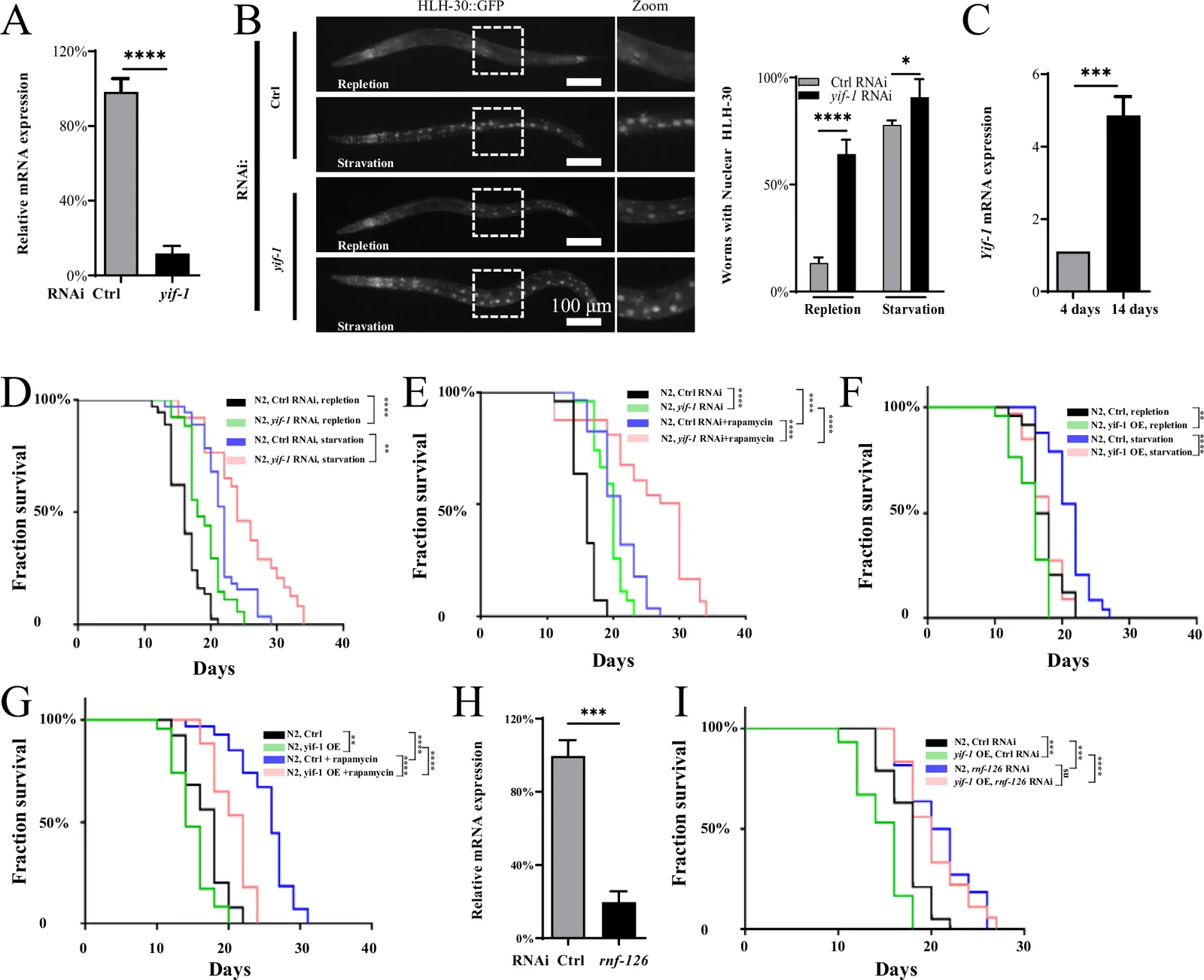
yif-1 has a conserved role in *C. elegans.* **A** RNAi knockdown efficiency of *yif-1* in the indicated worms. Data are expressed as mean ± SD (*n* = 3 biologically independent repeats). **B** HLH-30::GFP worms fed with Ctrl RNAi or RNAi against *yif-1* under starvation and repletion. Nuclear localization of HLH-30 was visualized. Inserts show enlarged intestinal cells. Quantification of the percentage of worms with HLH-30 in the nuclei of intestinal cells (*n* = 3 biologically independent repeats, ~ 40 worms per replicate). **C** Age-dependent changes in transcription levels of *yif-1* (*n* = 3 biologically independent repeats). **D–G** Lifespan analyses in the indicated strains and treatments. The data were obtained from 3 biologically independent experiments and analyzed using the log-rank (Mantel–Cox) test. **H** RNAi knockdown efficiency of *rnf-126* in the indicated worms. Data are expressed as mean ± SD (*n* = 3 biologically independent repeats). **I** Lifespan analyses in the indicated strains. The data were obtained from 3 biologically independent experiments and analyzed using the log-rank (Mantel–Cox) test. ns: not significant; *P ≤ 0.05; **P ≤ 0.01; ***P ≤ 0.001; ****P < 0.0001.

To determine the evolutionarily conserved role of YIF1A in mTORC1 signaling pathway, we knocked down *yif-1* in *C. elegans* expressing HLH-30::GFP (Fig. 7A) and assessed the effect of this depletion on nuclear translocation of HLH-30, the functional orthologue of mammalian TFEB. TFEB, a direct downstream substrate of mTORC1, is phosphorylated by mTORC1 at several serine residues (including Ser122, Ser142, and Ser211) under nutrient-replete conditions [54–56], and phosphorylation of these sites promotes TFEB retention in the cytosol [55, 57]. When not phosphorylated at these serines, TFEB accumulates in the nucleus [58]. Consistent with previous reports [59], HLH-30::GFP exhibited diffuse cytoplasmic localization under nutrient-rich conditions but accumulated in the nucleus upon starvation in control siRNA-treated worms (Fig. 7B). Strikingly, *yif-1* knockdown enhanced HLH-30::GFP nuclear localization under both fed and starved conditions (Fig. 7B), suggesting a regulatory interaction between yif-1 and mTORC1-dependent TFEB/HLH-30 trafficking.

Given mTORC1’s established role in lifespan regulation in *C. elegans* [59], we next investigated yif-1’s function in aging. Transcriptional profiling revealed age-dependent upregulation of *yif-1* (Fig. 7C). Genetic depletion of *yif-1* significantly extended lifespan under both fed and starved conditions, compared to wild-type controls (Fig. 7D). Furthermore, rapamycin further enhanced longevity in *yif-1* knocked down worms (Fig. 7E). Conversely, yif-1 overexpression significantly shortened lifespan (Fig. 7F), an effect reversed by rapamycin or *rnf-126* knocking down (Fig. 7G–I). Collectively, these findings demonstrate a conserved role for YIF1A in modulating mTORC1 signaling and longevity pathways.

## Discussion

The mechanistic target of rapamycin complex 1 (mTORC1), a central metabolic integrator, coordinates cellular growth and metabolic reprogramming in response to environmental cues [10]. Our study elucidates a previously unrecognized proteostatic mechanism regulating mTORC1 activity at the Golgi apparatus interface. We demonstrate that YIF1A potentiates mTORC1 signaling by facilitating RNF126-mediated K48-linked ubiquitination of G3BP1/2, ultimately driving their proteasomal degradation (Fig. S8M). These findings establish YIF1A as a critical spatial regulator of growth factor-dependent mTORC1 activation at the Golgi apparatus, directly linking organelle-specific protein quality control to metabolic signaling.

G3BP1/2 interacts with the TSC complex to suppress mTORC1 activity by inhibiting Rheb GTPase activity [10]. TSC1/2 have been reported to undergo post-translational modifications, such as ubiquitination and phosphorylation, to regulate mTORC1 activity [60–64]. In this study, we demonstrate that the ubiquitination of G3BP1/2 regulates mTORC1 signaling. Insulin stimulation triggered K48-linked ubiquitination of G3BP1/2, which attenuated TSC1/2-mediated inhibition of Rheb and coincided with the proteasomal degradation of both G3BP1/2 and TSC1/2, a process blocked by the proteasome inhibitor MG132. These findings reveal the critical role of G3BP1/2 ubiquitination in regulating mTORC1 signaling and enhance our understanding of the coordinated regulatory mechanisms involving the TSC complex.

In this study, we find that growth factor stimulation upregulates YIF1A protein levels and enhances mTORC1 activity, whereas YIF1A knockdown markedly attenuates mTORC1 activity, suggesting that the protein level of YIF1A is critical for mTORC1 activation. YIF1A promotes mTORC1 activity by interacting with RNF126 to mediate the ubiquitination of G3BP1/2. Upon serum starvation, YIF1A interacts with RNF126, and this interaction shows no significant change upon insulin stimulation. Importantly, insulin stimulation markedly increases YIF1A protein levels. Therefore, YIF1A may enhance its interaction by increasing its own protein level upon insulin stimulation, even though the change in interaction is not statistically significant. Notably, insulin stimulation has little effect on YIF1A transcription. However, its protein level is significantly increased by MG132, an inhibitor of the ubiquitin-proteasome pathway. These findings suggest the involvement of an E3 ubiquitin ligase or deubiquitinating enzyme in regulating YIF1A protein stability. Additionally, YIF1A protein levels are also increased in senescent tissues from mouse and senescent cell models, accompanied by enhanced mTORC1 activity. Notably, its deficiency significantly attenuates mTORC1 activity and correspondingly reduces cellular senescence. Therefore, the increased YIF1A contributes to mTORC1 activation to induce cellular senescence.

mTORC1 is a well-established regulator of cellular senescence [23], and its activity is persistently activated in senescent cells [23]. Subsequently, the persistently activated mTORC1 results in accumulation of p53 by increasing p53 synthesis and enhancing its stabilization mediated by inactivation of MDM2 [24], whereas p53 can promote the translation of p21, a cyclin-dependent kinase (CDK) inhibitor, and subsequently lead to cell cycle arrest. Additionally, sustained mTORC1 activity can trigger senescence by increasing transcriptional DNA damage [25], as evidenced by the enhanced γH2AX. Moreover, phosphorylation of S6 kinase by mTORC1 leads to the phosphorylation of Zrf1 [26], subsequently up-regulating p16^INK4A^ and resulting in cell cycle arrest. Therefore, we believe that YIF1A drives senescence at least in part by sustaining mTORC1 activity, which in turn promotes cell cycle arrest through downstream effectors such as γH2AX and p16^INK4A^. In support of this, we observed elevated p16^INK4A^ protein levels in aged mouse tissues, consistent with the enhanced YIF1A-mTORC1 signaling in these samples. Additionally, we also observed that YIF1A overexpression enhanced mTORC1 activity and γH2AX or p16^INK4A^ in senescent cells, whereas the opposite results were obtained in YIF1A knockdown cells. Therefore, we believe that the YIF1A-mTORC1 axis plays an important role in cellular senescence by targeting cell cycle arrest.

Accumulating evidence positions the Golgi apparatus as a compartmentally distinct mTORC1 regulator, capable of activating mTORC1 independently of lysosomal signaling [14]. This organelle’s architectural integrity directly modulates mTORC1 activity [14], with amino acids converging on Golgi-localized mTORC1 pools [16, 65]. Our findings reveal that the Golgi-resident protein YIF1A acts as a critical amplifier of growth factor-dependent mTORC1 activation. The small GTPase Rab1A, a key ER-Golgi apparatus trafficking regulator, is known to potentiate mTORC1 activity alongside other vesicular regulators (Rab5, ARF1) [16, 17]. Given YIF1A’s established role in ER-Golgi cargo shuttling [30], we propose a model where YIF1A spatially coordinates mTORC1 activation with secretory flux, potentially synchronizing anabolic signaling with biosynthetic demand. This mechanistic coupling between organelle trafficking and nutrient sensing may underlie the Golgi’s emerging role as a nutrient-integrative hub, though the precise molecular crosstalk requires further elucidation.

There is increasing evidence that the Yip1 domain family (YIPF) proteins have multifaceted functions in human pathophysiology. YIPF5 mutations are associated with developmental disorders including neonatal diabetes and microcephaly [66], yet paradoxically confer protection against DNA viral infections via STING-dependent innate immune regulation [67]. In the context of cancer, YIPF2 has been found to promote hepatocellular carcinoma (HCC) progression by facilitating the endocytic recycling of CD147 [68]. The functional dichotomy extends to YIF1 paralogs: whereas YIF1B contributes to both progressive encephalopathy and tumorigenesis [69–71], its homolog YIF1A has been implicated in cellular aging through poorly defined mechanisms [32–36]. Our study systematically deciphers YIF1A’s mechanistic involvement in cellular senescence. Quantitative analysis revealed selective upregulation of YIF1A protein levels during senescence induction, contrasting with stable YIF1B expression. Notably, the pro-senescent effects of YIF1A overexpression were effectively counteracted by rapamycin, suggesting mediation by the mTORC1 pathway. Evolutionary conservation of this mechanism was evidenced by lifespan extension in *C.elegans* following *yif-1* knockdown. As a central metabolic hub, the mTORC1 signaling pathway integrates diverse environmental stimuli, such as growth factors and amino acids, under regulation by upstream signaling proteins [3, 4, 62, 72–77]. Therefore, the mTOR inhibitor rapamycin can suppress mTORC1 activation mediated by these upstream regulators, including YIF1A. Consequently, rapamycin treatment further extended the lifespan of *C. elegans* with *yif-1* knockdown or overexpression, strongly suggests that yif-1 operates upstream of mTOR. Recent findings by McHugh et al. have demonstrated that genetic or pharmacological inhibition of the coatomer complex I (COPI) signaling pathway can selectively eliminate senescent cells [78], further underscores the Golgi apparatus as a critical signaling hub. Our data establish YIF1A as a novel regulator of mTORC1-mediated senescence through Golgi apparatus-dependent signaling cascades, proposing a unified model where Golgi-derived signals coordinate cellular aging processes.

In summary, our study identified the role of YIF1A-mTORC1 signaling pathway in cellular aging, which further determined Golgi acting as important signaling hub in cells. These findings highlight YIF1A-mTORC1 as a potential target for the development of anti-aging strategies.

## Materials and methods

### Cells culture and plasmids

The following human cell lines were used in this study: Sum159PT (RRID: CVCL_5423), MDA-MB-231 (RRID: CVCL_0062), NCI-H1299 (RRID: CVCL_0060) and HEK293T (RRID: CVCL_0063) from Cellverse (China); MCF7 (RRID: CVCL_0031) from KeyGENE Biotech (China); IMR-90 (RRID: CVCL_0347) from Whelab (China) and HeLa (RRID: CVCL_0030) from Servicebio (China). All cells were cultured at 37 °C with 5% CO_2_ atmosphere in the appropriate medium (DMEM for Sum159PT, HeLa, HEK293T and MDA-MB-231; RPMI 1640 for MCF7 and NCI-H1299; MEM for IMR-90) containing 10% FCS. All cell lines used in this study were routinely screened for mycoplasma contamination using the Mycoplasma Detection Kit (Servicebio, China), with results confirming the absence of mycoplasma in all cultures. The mTOR signaling pathway is highly conserved across cell types. The cell lines utilized in our study are well-established and commonly used models in biomedical research.

*YIF1A* (Accession number: CR456710), *RNF126* (Accession number: BC025374), *G3BP1* (Accession number: U32519) and *G3BP2*(Accession number: AF145284) were cloned from HEK293T cDNA and ligated to related plasmids. HA-ubiquitin, HA-ubiqutin K48 and HA-ubiqutin K63 plasmid were stored by my laboratory. HA-ubiquitin K48 and HA-ubiquitin K63 denote ubiquitin variants in which the 48th or 63rd amino acid of wild-type ubiquitin is K, while other lysines were mutated to arginines [53].

### Strains of C. elegans

The *C.elegans* strains used in this study include N2 Bristol (wild type) and HLH-30::GFP (MAH235). All of these strains were provided by the Caenorhabditis Genetic Center (CGC, University of Minnesota, USA). All worm strains were maintained on solid nematode growth media (NGM) using *E. coli* OP50 unless otherwise indicated. All worm strains were kept at 20 °C.

### Chemicals, antibodies and beads

The following regents and antibodies were used in this study: insulin (HY-P0035, MCE), epidermal growth factor (EGF) (HY-P7109, MCE), etoposide (ETO) (HY-13629, MCE), doxorubicin (DOX) (HY-15142A, MCE), MG132 (474790, Merk), bafilomycin A1(HY-100558, MCE), brefeldin A (HY-16592, MCE), ZSTK474 (MCE, HY-50847), anti-GFP (M20004, Abmart), anti-Flag antibody (M20008, Abmart), anti-β-actin (AC006, Abclone), anti-β-tubulin(M20005, Abmart), anti-HA (sc-57592, Santa Cruz), anti-HA (3724, CST), anti-GAPDH (M20006, Abmart), anti-TSC1(sc-377387, Santa Cruz), anti-TSC2 (4308, CST), anti-G3BP1(sc365338, Santa Cruz), anti-G3BP2 (16276-1-AP, Proteintech), anti-Rheb (sc271509, Santa Cruz), anti-Lamp1(15665, CST), anti-Lamp1(9091, CST), anti-RNF126 (66647-1-I, Proteintech), anti-YIF1A (TA333737, ORIGENE), anti-YIF1A (PA5140186, Thermo), anti-P16^INK4A^ (SC1661, Santa Cruz), anti-γ-H2AX (Ser139) (sc-517348, Santa Cruz), anti-Tim23 (sc-514463, Santa Cruz), anti-Calnexin (66903-1-Ig, Proteintech), anti-ubiquitin (AF1705, Beyotime). The M2 Flag beads, HA beads, Protein A/G beads and GFP(A) beads were purchased from MCE, Beyotime, Selleck and Abmart, respectively.

### Insulin and EGF treatments

For insulin and EGF treatment, the cells were starved of serum for 50 min, and starved or re-stimulated with 100 nM insulin or 20 nM EGF for 10 or 15 min. For insulin treatment at HEK293T cells, cells were starved of serum for 15 h, or starved or re-stimulated with 100 nM insulin for 15 min. For inhibitory assays, the cells were pre-incubated with 100 nM rapamycin for 50 min, 100 nm ZSTK474 for 50 min and 2 μg/ml brefeldin A for 8 h before addition of 100 nM insulin.

### cDNA transfection or gene knockdown in cells

Plasmids were transfected into cells with polyethylenimine (PEI) according to the instruction. After transfection for 6 h, the culture medium was replaced with fresh cell culture medium supplemented with 10% FCS. HA-ubiquitin K48 and HA-ubiquitin K63 denote ubiquitin variants in which the 48th or 63rd amino acid of wild-type ubiquitin is K, while other lysines were mutated to arginines [53].

To construct the stable knocking down cell lines, the encoding shRNA plasmids were co-transfected with psPAX2 and PMD2.G in HEK293T cells and further cultured for 48 h. The collected supernatants were filtered with 0.45 μm filterable membrane and added to the cultured cells. The infected cells were then screened with puromycin until the single-cell colonies were formed. The shRNA sequences used in this study were synthesized as follows: TTCTCCGAACGTGTCACGT for negative control (NC); GTCTGTGAGCAAACTCAAGTA for *YIF1A*; TGCCATCATCACACAGC TCCT for *RNF126*.

### Immunoblotting

The treated cells were lysed with 1×SDS loading buffer, and then boiled at 100 °C for 10 min. The lysate were subjected to SDS-PAGE and transferred to PVDF membrane. After blocking with 10% skimmed milk in TBST, the PVDF membranes were incubated overnight with primary antibodies at 4 °C, followed by incubation with HRP-conjugated secondary antibodies for 2 h at room temperature. The membranes were visualized using a chemiluminescence imaging system (Tanon).

### Immunoprecipitation (IP)

The HEK293T cells were collected by centrifugation at 1 000 rpm for 5 min, and then rinsed with cold PBS. The collected cells were lysed with NETN lysis buffer (100 mM NaCl, 1 mM EDTA, 20 mM Tris-HCl, pH 8.0, 0.5% NP-40) containing protease inhibitor (Sellck) for 30 min at 4 °C. The lysates were centrifuged at 17 000 g for 10 min to separate cell debris and supernatant. The cell supernatant was incubated with GFP or M2 Flag beads at 4 °C for 3 h. After being centrifuged at 3 000 g for 2 min, the collected beads were washed three times with NETN lysis buffer, and then eluted with 1×SDS loading buffer at 100 °C for 5 min.

### Detection of protein ubiquitination

A denaturing IP was performed to detect the ubiquitination of G3BP1/2. Briefly, collected cells were lysed in 1×SDS loading buffer, boiled for 10 min, and subsequently diluted with 10-fold volume of NETN lysis buffer. The diluted lysates were incubated with GFP beads at 4 °C for 4 h. After incubation, the beads were collected by centrifugation, washed three times with NETN lysis buffer, and finally eluted with 1× SDS loading buffer. For native IP of ubiquitinated G3BP1/2, the boiled lysates were incubated with anti-G3BP1/2 antibodies or isotype control antibodies at 4 °C for 4 h, followed by incubation with Protein A/G beads to isolate endogenous G3BP1/2.

### Immunofluorescence

The cells were seeded in 6-well plates containing 14 mm coverslips. After treated with corresponding compound, the cells were fixed with 4% paraformaldehyde (PFA) for 10 min at room temperature and then rinsed with PBS three times. The fixed cells were permeabilized with 0.3% Trition X-100 in PBS for 5 min, and then rinsed with PBS three times. After blocking with 3% BSA in PBS for 30 min at 37 °C, the permeabilized cells were sequential incubated with related primary antibodies for whole night at 4 °C and secondary antibodies conjugating with related fluorescein. After staining DAPI for nuclear, the coverslips were sealed, and then observed with confocal microscopes (LEICA TCS SPE). All experiments about the co-localization were quantified using Fiji software (version 1.0).

### Identification of YIF1A-binding proteins by mass spectrometry

HEK293T cells expressing Flag-YIF1A were collected to perform an IP assay with Flag magnetic beads. After being collected at 2 000 g and washed with NETN buffer three times, beads were incubated with 100 μl 3× Flag Peptide (150 μg/ml) at 4 °C for 2 h to elute the binding proteins. The eluents were then mixed with 20 μl 5×SDS loading buffer, boiled at 100 °C for 10 min, resolved by SDS-PAGE and then stained with Coomassie G-250 staining. After destaining with 50% ethanol, the gels were dehydrated in 100% acetonitrile (ACN), and reduced with 10 mM dithiothreitol (DTT) at 56 °C for 1 h. The gel was dried with ACN and then alkylated with 55 mM iodoacetamide (IAA) in the dark for 1 h. After being dehydrated with ACN, the gels were digested with trypsin (Promega) at 37 °C overnight and added 5% trifluoroacetic Acid (TFA) and 50% CAN to extract peptides with sonication. Finally, the extracted peptides were dried and analyzed using SCIEX Triple TOF 5600 LC-MS/MS.

### GST pull-down

Rosetta-gami2 (DE3) *E. coli* cultures expressing GST-YIF1A or empty GST vector were grown at 37 °C until reaching an optical density (OD) of 0.6, and then cooled to 25 °C. The cultures were then induced with 0.5 μM IPTG at 25 °C overnight to induce protein expression. The bacteria were pelleted at 6000 RPM for 15 min, resuspended in lysis buffer (137 mM NaCl, 2.7 mM KCl,10 mM Na_2_HPO_4_, 1.8 mM KH_2_PO_4_, 1 mM PMSF, 2 mM EDTA, 1 mM DTT) and then lysed by sonication (SCIENTZ-IID, SCIENTZ). The lysates were centrifuged (12000 rpm, 20 min, 4 °C) to collect supernatant, and supernatants were incubated with glutathione Sepharose beads to collect the target proteins by rotating at 4 °C for 2 h.

For binding assays, the purified GST or GST-bound YIF1A proteins were incubated with HEK29T cell lysates at 4 °C for 3 h. Beads were pelleted at 4 °C, washed with cell lysis buffer for 3 times and then were eluted with 1×SDS loading buffer by boiling at 95 °C for 10 min.

### Golgi-IP

Golgi apparatus were purified as previously described [46]. Briefly, HEK29T cells stabling expressing TMEM115-HA were collected and homogenized in isotonic potassium-supplemented phosphate-buffer saline (KPBS) (136 mM KCl, 10 mM KH_2_PO_4_, pH 7.25) using glass Dounce homogenizer. Homogenates were centrifuged at 1000 × *g* for 2 min at 4 °C to clear off debris and the resulting supernatants containing the cellular organelles were incubated with HA magnetic beads for 5 min at 4 °C. Then, the beads were washed 3 times with cold KPBS and eluted with 1×SDS loading buffer by boiling at 95 °C for 10 min.

### mRNA quantifications by Real-time PCR

Cells or worms were lysed with TRIzol reagent (Takara) and the total RNAs were then extracted by the standard chloroform extraction and isopropanol precipitation procedure. Real-time PCR was performed using HiScript® II One Step qRTQrt-PCR SYBR® Green KIT (Vazyme) on QuantStudio 3 (Thermofisher). Relative mRNA levels of target genes were normalized against the geometric mean of the housekeeping genes. Mammalian cell transcript quantifications were normalized to *18S*, and *C. elegans* transcript quantifications were normalized to *act-1*. The primers used were as follows:

*YIF1A* forward: ATGGCTTATCACTCGGGCTAC

*YIF1A* reverse: CCGCTTGTGTCATCGAAGAGG

*RNF126* forward: GGAAGAGACCAGGAGCACAGAA

*RNF126* reverse: GATGCCGAAAGCAAACTGTCCG

*G3BP1* forward: CGGGCGGGAATTTGTGAGA

*G3BP1* reverse: TCTGTCCGTAGACTGCATCTG

*G3BP2* forward: GTAGGGCGGGAGTTTGTGAG

*G3BP2* reverse: CTGGGGCTTTCCACTAGCATC

*TSC1* forward: CAACAAGCAAATGTCGGGGAG

*TSC1* reverse: CATAGGGCCACGGTCAGAA

*TSC2* forward: CCAAACCAACAAGCAAAGATTCA

*TSC2* reverse: CACATTCCATGCTCAGTTCTCT

*Rheb* forward: TTGTGGACTCCTACGATCCAA

*Rheb* reverse: GGCTGTGTCTACAAGTTGAAGAT

*18S* forward: GGAGTATGGTTGCAAAGCTGA

*18S* reverse: ATCTGTCAATCCTGTCCGTGT

*yif-1* forward:TGATTCTTGTGGCGTTTGTAGTCA

*yif-1* reverse: TACCATTCGCGGAGCAGTTGA

*rnf-126* forward: TGAGCCGTGGCTTAAGACTAA

*rnf-126* reverse: GATCAGCTTCCTGGAGCTTTTT

*act-1* forward: TCGGTATGGGACAGAAGGAC

*act-1* reverse: CATCCCAGTTGGTGACGATA

### Induction of cellular senescence

HEK 293 T cells were treatedwith 10 μM etoposide (ETO) or 4 μM doxorubicin (DOX) for 24 h and released for two or three days. MCF7 cells were treated with 2 or 10 μM ETO for 7 or 4 days, respectively. MCF7 cells were treated with 4 or 10 μM DOX for 7 or 4 days, respectively. Additionally, MCF7 cells were treated with 10 μM ETO for 24 h and released for 4, 5, 6 and 7 days. 100 nM rapamycin or 100 ng/ml Brefeldin A (BFA) were added to cell medium following the induction of cellular senescence with indicated compounds.

To establish replicative senescence, IMR90 cells at population doubling (PD) 32 were serially passaged until PD 88.

### SA-β-gal staining

Cells were seeded in six-well dishes and then exposured to indicated compounds. Then, cells were fixed and stained using Cell Senescence β-Galactosidase Staining Kit (Biosharp, BL133A) according to the instruction.

### RNAi in worms

RNAi experiments were performed using the feeding method. The *yif-1*(F57A8.2) RNAi bacterial clone was obtained from Dharmacon©. The *rnf-126* RNAi construct was generated by cloning a gene-specific fragment from the *rnf-126* (Y54E10BR.3) coding sequence into the L4440 vector. The sense strand sequences of the cloned fragment for expressing dsRNAs is: *rnf-126*: 5’-*ACAGGGAGCACCACAAG AAC*…(to)…*AGGATGTTGGTGCACTCGAC*-3’. The recombinant L4440-*rnf-126* plasmid or the empty L4440 vector (control) was transformed into the RNase III-deficient *E. coli* HT115(DE3) strain for double-stranded RNA expression.

For RNAi feeding, HT115 strains harboring the indicated RNAi vectors or the empty L4440 control were grown overnight at 37 °C on LB plates supplemented with tetracycline (15 μg/mL) and carbenicillin (2 mg/mL). A single colony was inoculated into LB broth containing carbenicillin (500 μg/mL) and incubated for 8 h at 37 °C with shaking. Bacterial lawns were then spread onto NGM plates containing 1 mM IPTG and 25 μg/mL carbenicillin and induced for 12 h at 37 °C. Synchronized L1-stage worms were placed onto these plates and fed the RNAi bacteria for 40 h until they reached the L4 stage, at which point they were harvested for subsequent treatments. RNAi knockdown efficiency was confirmed by quantitative real-time PCR.

### Construction of the stable transgenic *yif-1* OE strain

To generate a stable overexpression strain of the *yif-1* gene in *C. elegans*, we employed a plasmid-based microinjection approach, followed by genomic integration via X-ray irradiation. Microinjections were performed using the transgene plasmid Prpl-28-*yif-1*-GFP-unc-54 3’ UTR at a concentration of 5 ng/μL, co-injected with empty vector pSL1190 plasmid and Pmyo-2::mCherry marker into wild-type N2 worms. As reporter marker, Pmyo-2::mCherry was used to visually confirm transgene presence. Following microinjection, worms carrying extrachromosomal arrays were exposed to X-ray irradiation to integrate the transgene into the genome. Stable integrated lines were isolated by screening for consistent mCherry expression in the pharynx, indicating successful integration. Sunybiotech provided comprehensive support, including plasmid construction, microinjections, X-ray irradiation of the extrachromosomal lines, and the isolation of stable integrated strains.

### HLH-30::GFP nuclear translocation assay

Synchronized HLH-30::GFP worms (MAH235) were raised at 20 °C until the L4 stage. These worms were then subjected to either nutrient starvation or nutrient repletion for 4 h. Following treatment, the worms were mounted on 2% agarose pads and imaged for HLH-30::GFP localization using a Leica fluorescence dissecting microscope (LEICA TCS SPE) within 5 min. The experiment included three biological replicates, with 30 worms analyzed per replicate.

### *C. elegans* lifespan assays

Worms were raised on plates with the indicated RNAi bacteria (Fig. 7D, E) or OP50 bacteria (Fig. 7F, G) until day 7. OP50 or HT115 bacteria (worms’ food) at the concentration of 1 × 10^8^/ml and 1×10^11^/ml were used as starvation and repletion food, respectively. Nutrient restriction and repletion treatments were started at the end of the reproductive period (day 7) because starvation during the reproductive period would cause worm death. Worms from different treatment groups were randomly switched to fresh bacterial cultures for starvation or repletion every other day. A total of 40 worms per biological replicate were used for each group, and the sample size was determined by reference to the literature. Worms that did not respond to gentle touch were scored as dead. Animals that crawled off the plate or died owing to the vulva bursting were excluded. For the treatment with rapamycin, 50 mg/ml rapamycin dissolved in DMSO was added to plate agar to a final concentration 100 μM. Meanwhile, the appropriate DMSO concentration was added to plate agar as control plates (Fig. 7E, G). The investigators who collected the data were blinded to genotype and treatment. The significance of survival curves was determined by P values calculated using the log-rank (Mantel-Cox) test.

### Mouse experiments

3-month-old (Young, *n* = 7) and 24-month-old (Aged, *n* = 7) male C57BL/6 J mice were used in this study. All mice were housed in a barrier facility under 12-h dark/light cycles with free access to water and standard chow. The mice were euthanized at the indicated times and their tissues (spleen, lung, liver, heart and kidney) were harvested. Blinding was implemented in this experiment, and no randomization was performed for this experiment. All procedures were conducted in accordance with the “Guiding Principles in the Care and Use of Animals” (China) and were approved by the Laboratory Animal Ethics Committee of Binzhou Medical University.

### Statistical analysis

Student’s t-test was used to compare two experimental conditions (Figs. 1A, 2D–K, 3F, G, 4C–F, 4I-J, 5A, B, 5F, G, 5K, L, 7A, 7C, 7H; Figs. S1, S2I, S3F, G, S3K–N, S4A, S5F, G and S7C, D). For comparison of more than two conditions, a one-way ANOVA (Fig. 4G, H; S3I, J; S5B, C and S5F, G) and two-way ANOVA test (Figs.1B–H, 2A–C, 3H, 4A, B, 5C–E, 5H–J, 6, 7B; Figs. S2A, B, S3H, S4B–H, S5D-E, S6B–G, S7A, B, and S8A–L) was applied. The survival curves of *C. elegans* were calculated using the log-rank (Mantel–Cox) test (Fig.7D–G, 7I). All the statistical analyses and preparation of the columns in the Figures were performed using Graphpad Prism 9.0.

## Supplementary information

Supplementary Fig.s

Original Western blot
