# Supplementary material for: YIF1A activates mTORC1 signaling to promote cellular senescence
Source: Cell Death Dis. 2026 Jun 23;17(1):778. doi: 10.1038/s41419-026-09034-z (PMC13542221; doi:10.1038/s41419-026-09034-z)
Supplement: Supplementary file 1 [file 41419_2026_9034_MOESM2_ESM.pdf]

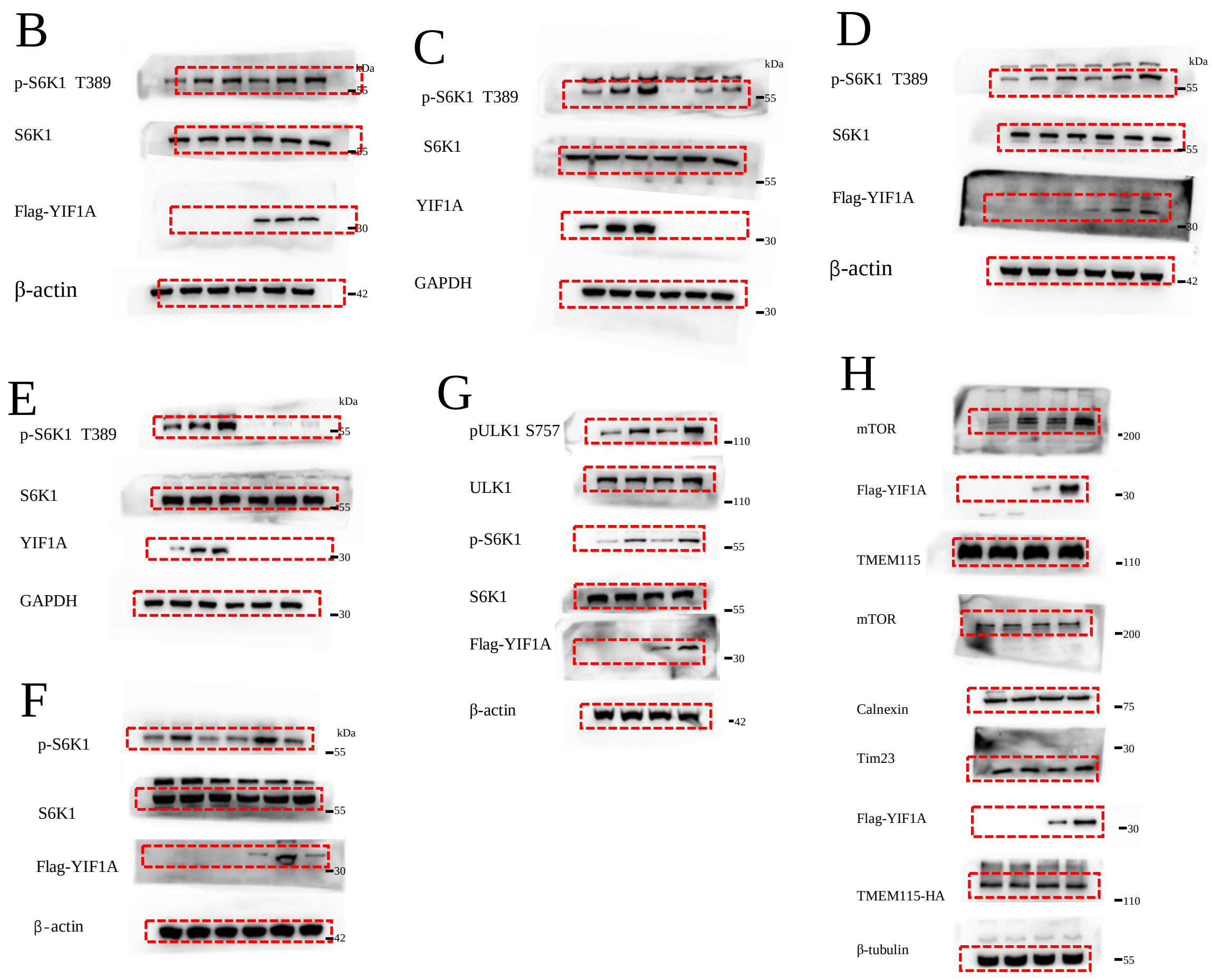
B
p-S6K1 T389
S6K1
Flag-YIF1A
β-actin
kDa
55
55
30
42
C
p-S6K1 T389
S6K1
YIF1A
GAPDH
kDa
55
55
30
30
D
p-S6K1 T389
S6K1
Flag-YIF1A
β-actin
kDa
55
55
30
42
E
p-S6K1 T389
S6K1
YIF1A
GAPDH
kDa
55
55
30
30
F
p-S6K1
S6K1
Flag-YIF1A
β-actin
kDa
55
55
30
42
G
pULK1 S757
ULK1
p-S6K1
S6K1
Flag-YIF1A
β-actin
110
110
55
55
30
42
H
mTOR
Flag-YIF1A
TMEM115
mTOR
Calnexin
Tim23
Flag-YIF1A
TMEM115-HA
β-tubulin
200
30
110
200
75
30
30
110
55

Fig. 1

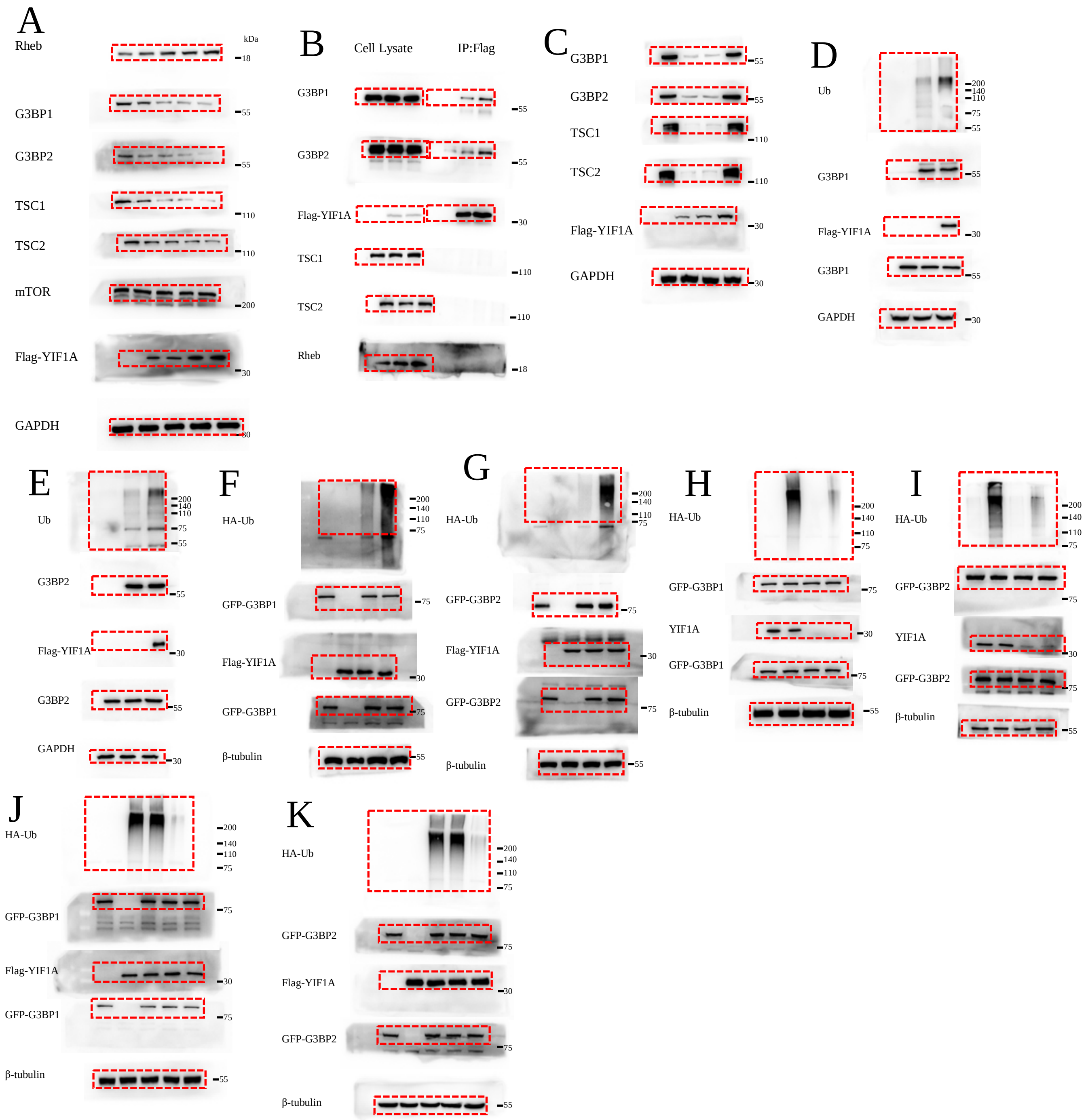
A
Rheb
kDa
G3BP1
G3BP2
TSC1
TSC2
mTOR
Flag-YIF1A
GAPDH
B
Cell Lysate
IP:Flag
G3BP1
G3BP2
Flag-YIF1A
TSC1
TSC2
Rheb
C
G3BP1
G3BP2
TSC1
TSC2
Flag-YIF1A
GAPDH
D
Ub
G3BP1
Flag-YIF1A
G3BP1
GAPDH
E
Ub
G3BP2
Flag-YIF1A
G3BP2
GAPDH
F
HA-Ub
GFP-G3BP1
Flag-YIF1A
GFP-G3BP1
β-tubulin
G
HA-Ub
GFP-G3BP2
Flag-YIF1A
GFP-G3BP2
β-tubulin
H
HA-Ub
GFP-G3BP1
YIF1A
GFP-G3BP1
β-tubulin
I
HA-Ub
GFP-G3BP2
YIF1A
GFP-G3BP2
β-tubulin
J
HA-Ub
GFP-G3BP1
Flag-YIF1A
GFP-G3BP1
β-tubulin
K
HA-Ub
GFP-G3BP2
Flag-YIF1A
GFP-G3BP2
β-tubulin

Fig. 2

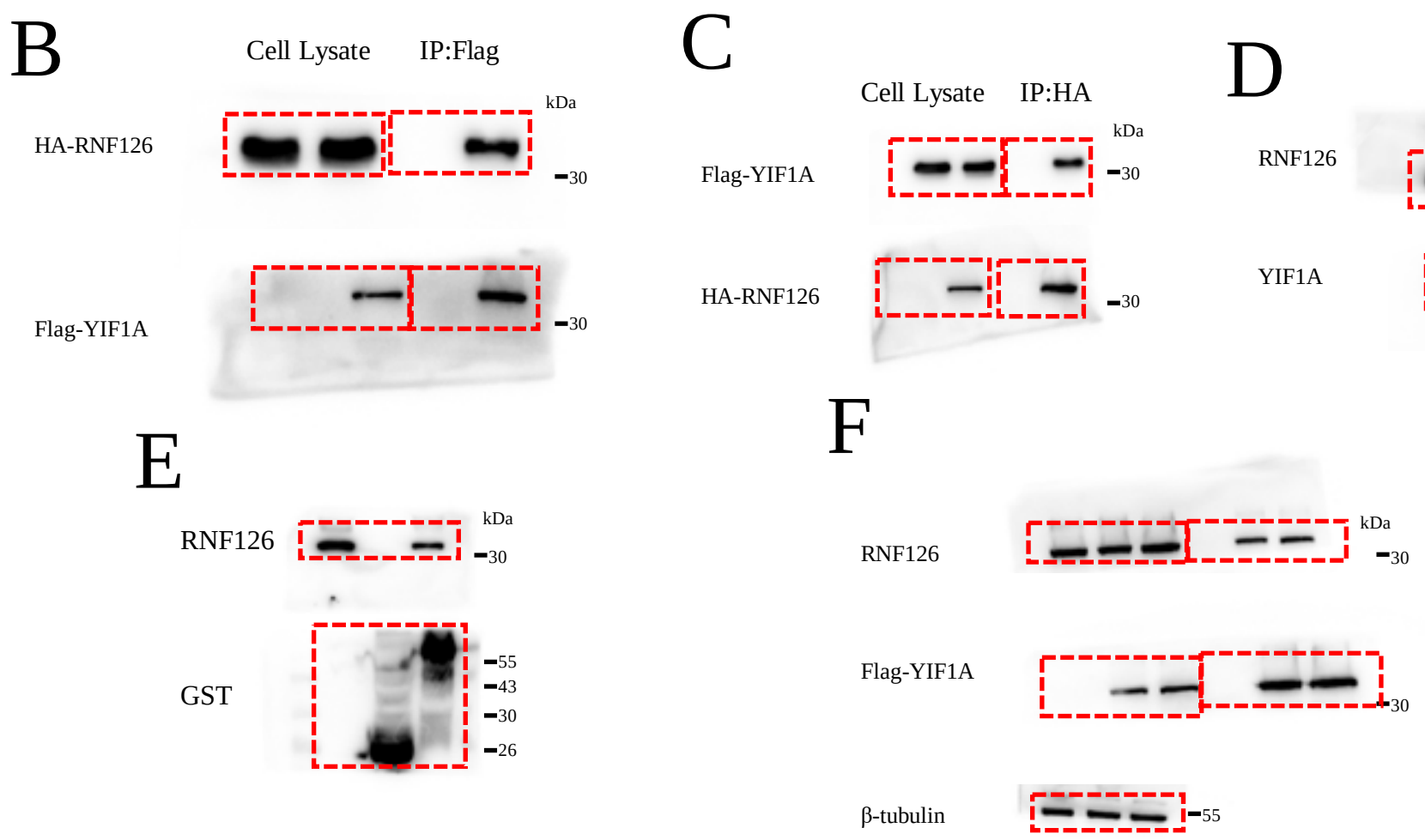
B
Cell Lysate
IP:Flag
kDa
HA-RNF126
Flag-YIF1A
C
Cell Lysate
IP:HA
kDa
Flag-YIF1A
HA-RNF126
D
kDa
RNF126
YIF1A
E
kDa
RNF126
GST
F
kDa
RNF126
Flag-YIF1A
β-tubulin

Fig. 3

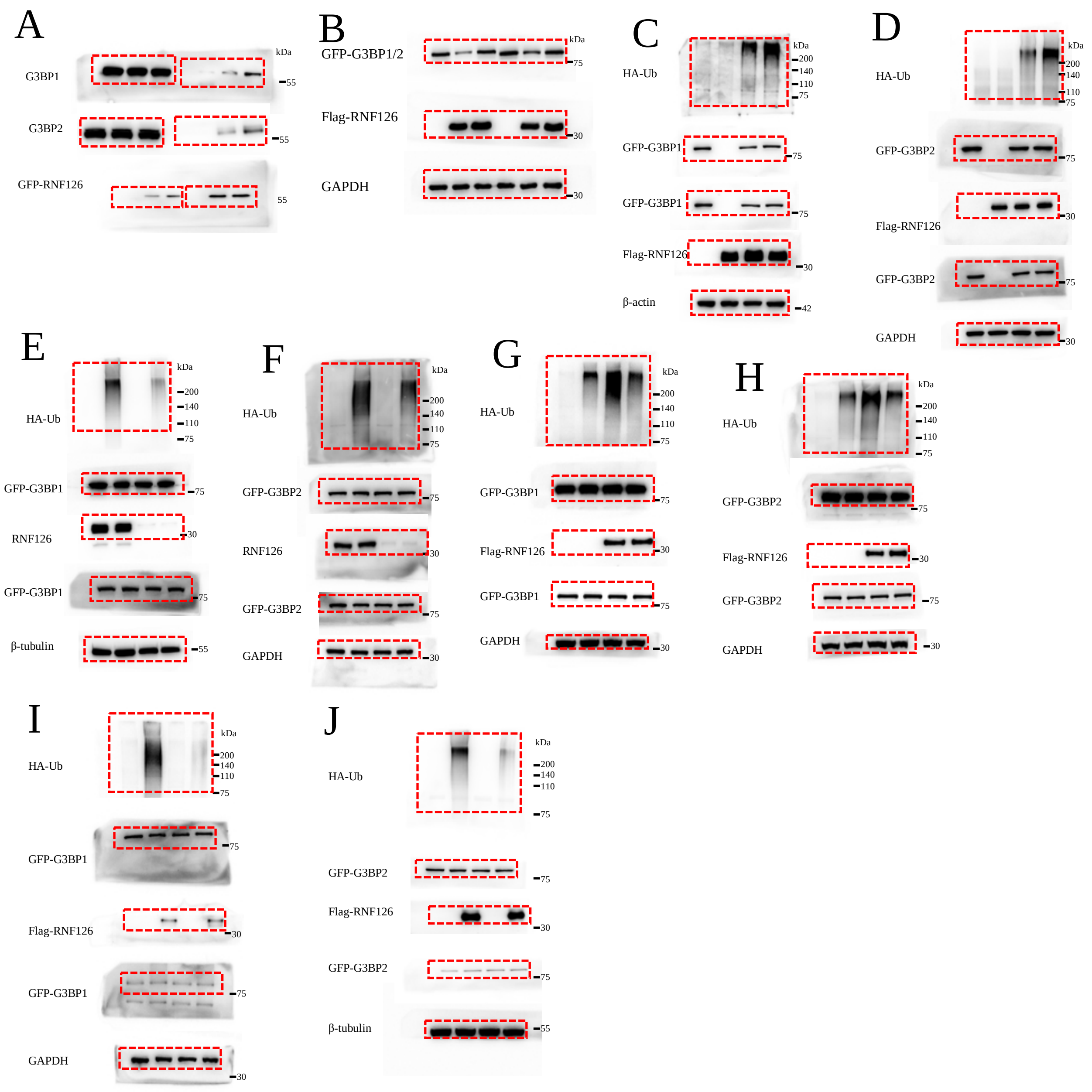
A
G3BP1
G3BP2
GFP-RNF126
kDa
55
B
GFP-G3BP1/2
Flag-RNF126
GAPDH
75
30
C
HA-Ub
GFP-G3BP1
Flag-RNF126
β-actin
200
140
110
42
D
HA-Ub
GFP-G3BP2
Flag-RNF126
E
GFP-G3BP1
RNF126
β-tubulin
F
G
H
I
J

Fig. 4

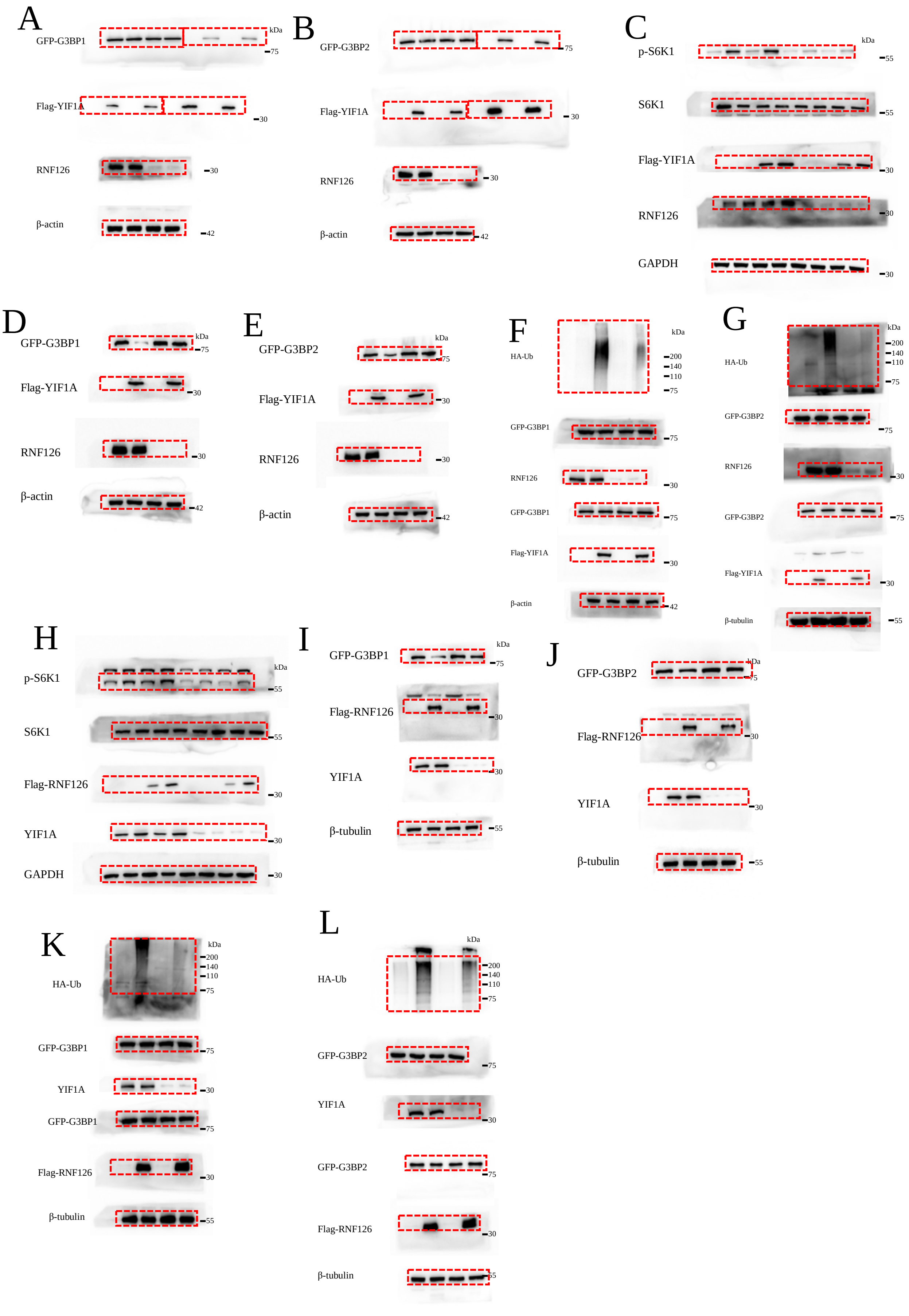
A
GFP-G3BP1
Flag-YIF1A
RNF126
β-actin
B
GFP-G3BP2
Flag-YIF1A
RNF126
β-actin
C
p-S6K1
S6K1
Flag-YIF1A
RNF126
GAPDH
D
GFP-G3BP1
Flag-YIF1A
RNF126
β-actin
E
GFP-G3BP2
Flag-YIF1A
RNF126
β-actin
F
HA-Ub
GFP-G3BP1
RNF126
GFP-G3BP1
Flag-YIF1A
β-actin
G
HA-Ub
GFP-G3BP2
RNF126
GFP-G3BP2
Flag-YIF1A
β-tubulin
H
p-S6K1
S6K1
Flag-RNF126
YIF1A
GAPDH
I
GFP-G3BP1
Flag-RNF126
YIF1A
β-tubulin
J
GFP-G3BP2
Flag-RNF126
YIF1A
β-tubulin
K
HA-Ub
GFP-G3BP1
YIF1A
GFP-G3BP1
Flag-RNF126
β-tubulin
L
HA-Ub
GFP-G3BP2
YIF1A
GFP-G3BP2
Flag-RNF126
β-tubulin
kDa

Fig. 5

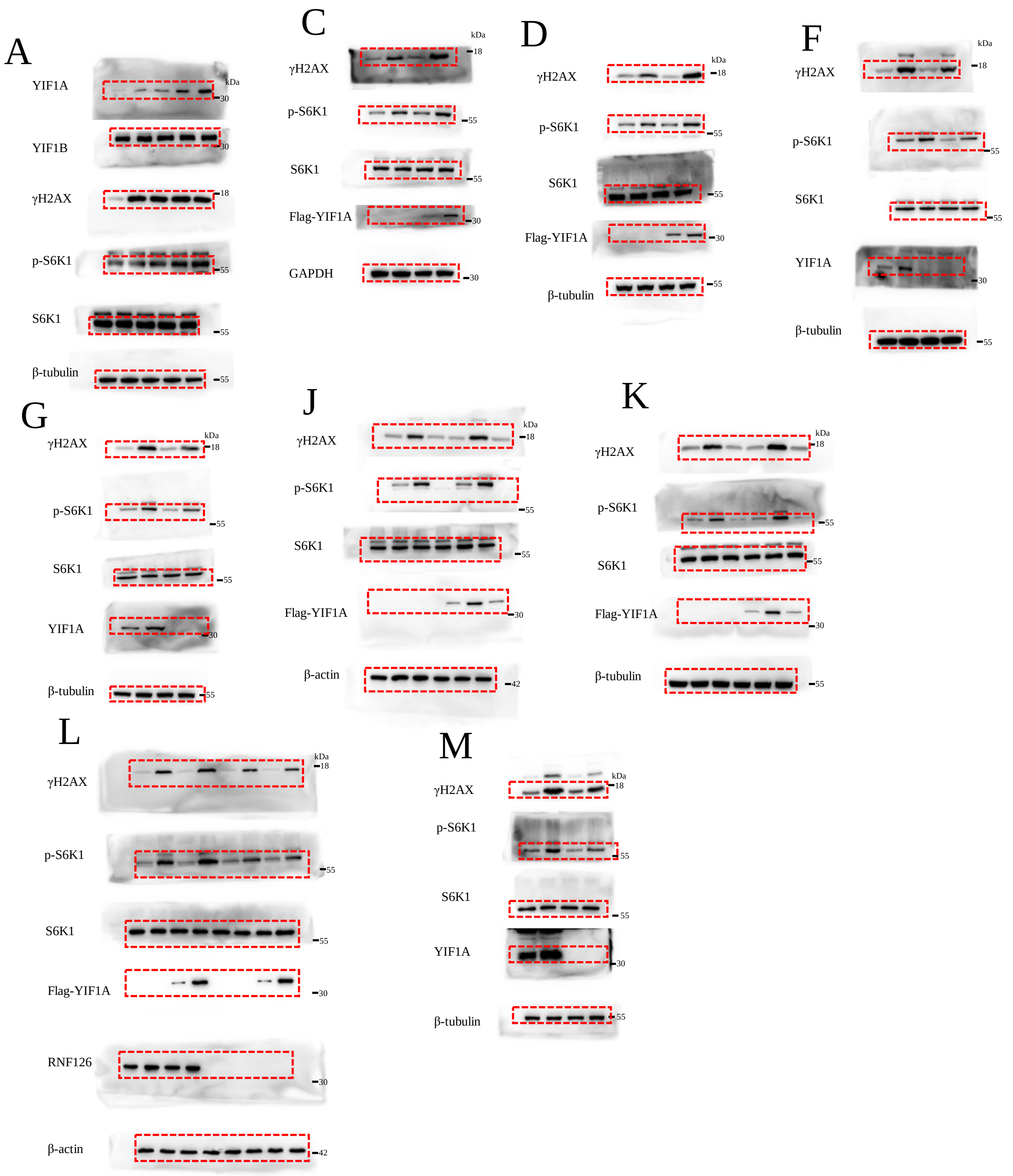
A
YIF1A
YIF1B
γH2AX
p-S6K1
S6K1
β-tubulin
kDa
30
30
18
55
55
55
C
γH2AX
p-S6K1
S6K1
Flag-YIF1A
GAPDH
kDa
18
55
55
30
30
D
γH2AX
p-S6K1
S6K1
Flag-YIF1A
β-tubulin
kDa
18
55
55
30
55
F
γH2AX
p-S6K1
S6K1
YIF1A
β-tubulin
kDa
18
55
55
30
55
G
γH2AX
p-S6K1
S6K1
YIF1A
β-tubulin
kDa
18
55
55
30
55
J
γH2AX
p-S6K1
S6K1
Flag-YIF1A
β-actin
kDa
18
55
55
30
42
K
γH2AX
p-S6K1
S6K1
Flag-YIF1A
β-tubulin
kDa
18
55
55
30
55
L
γH2AX
p-S6K1
S6K1
Flag-YIF1A
RNF126
β-actin
kDa
18
55
55
30
30
42
M
γH2AX
p-S6K1
S6K1
YIF1A
β-tubulin
kDa
18
55
55
30
55

Fig. 6

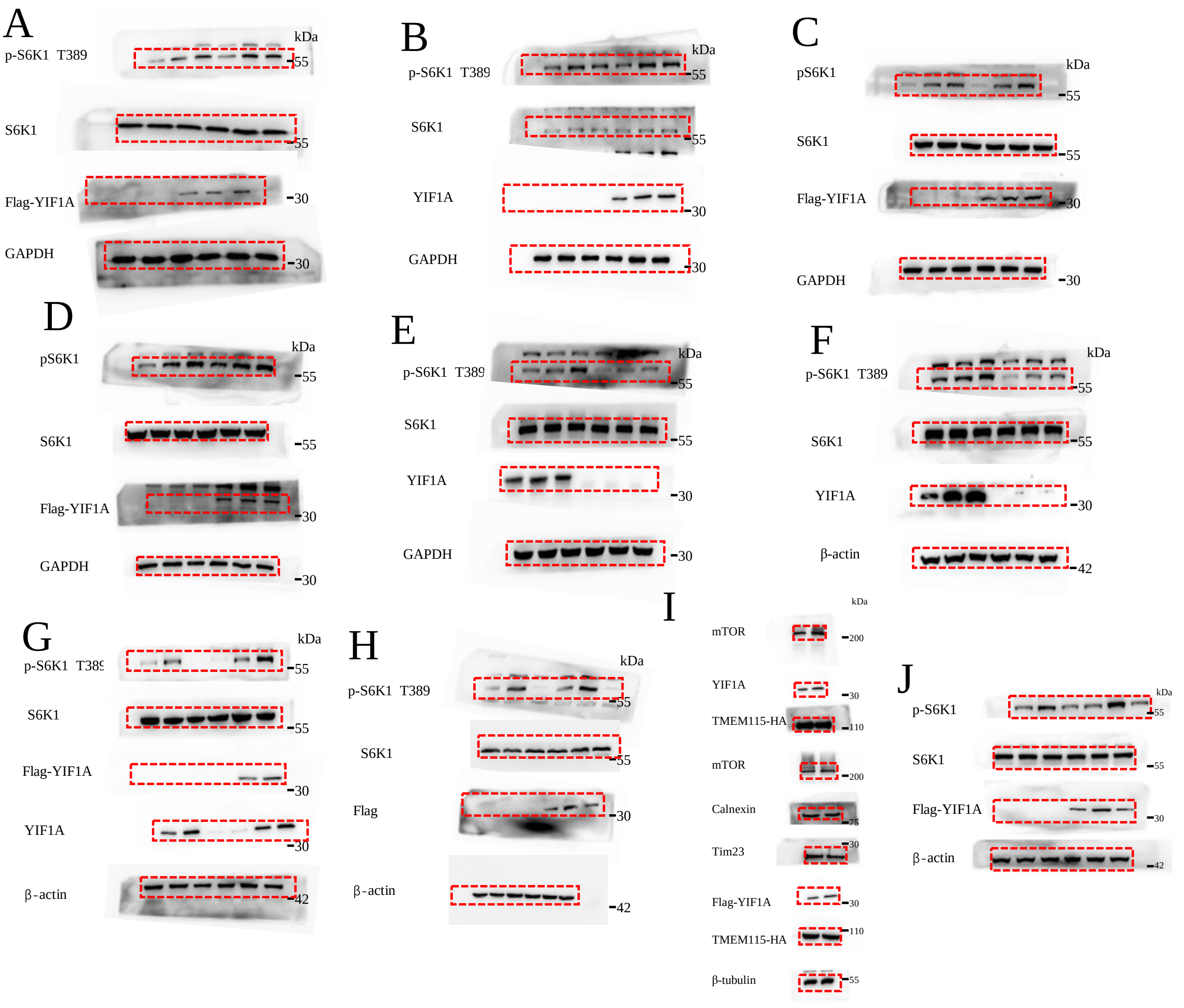
A
B
C
D
E
F
G
H
I
J
kDa
p-S6K1 T389
pS6K1
p-S6K1
S6K1
Flag-YIF1A
YIF1A
Flag
GAPDH
β-actin
mTOR
TMEM115-HA
Calnexin
Tim23
β-tubulin
55
30
42
200
110
75

Fig. S2

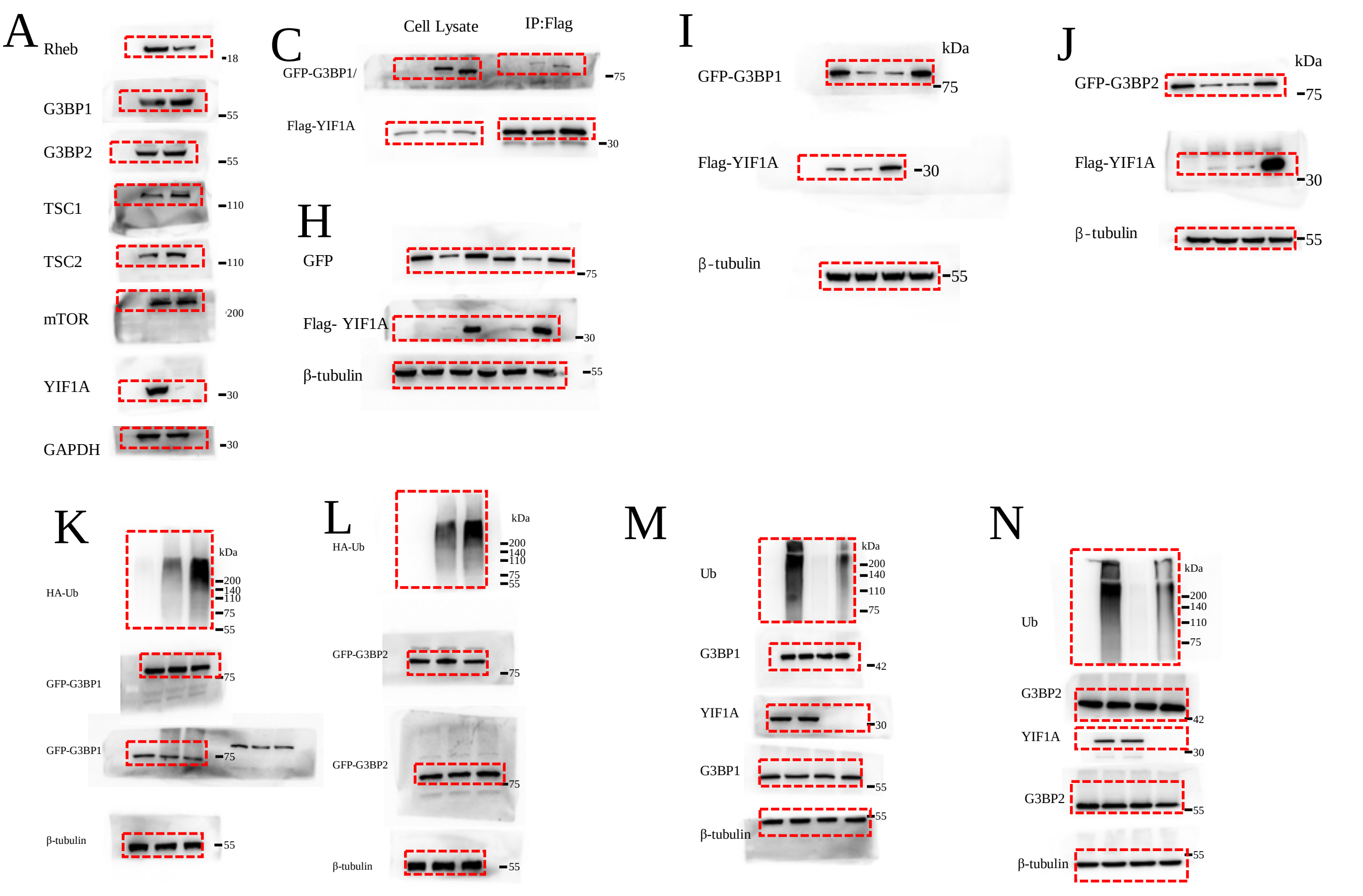
A
Rheb
G3BP1
G3BP2
TSC1
TSC2
mTOR
YIF1A
GAPDH
C
Cell Lysate
IP:Flag
GFP-G3BP1/
Flag-YIF1A
H
GFP
Flag- YIF1A
β-tubulin
I
kDa
GFP-G3BP1
Flag-YIF1A
β-tubulin
J
kDa
GFP-G3BP2
Flag-YIF1A
β-tubulin
K
kDa
HA-Ub
GFP-G3BP1
GFP-G3BP1
β-tubulin
L
kDa
HA-Ub
GFP-G3BP2
GFP-G3BP2
β-tubulin
M
kDa
Ub
G3BP1
YIF1A
G3BP1
β-tubulin
N
kDa
Ub
G3BP2
YIF1A
G3BP2
β-tubulin

Fig. S3

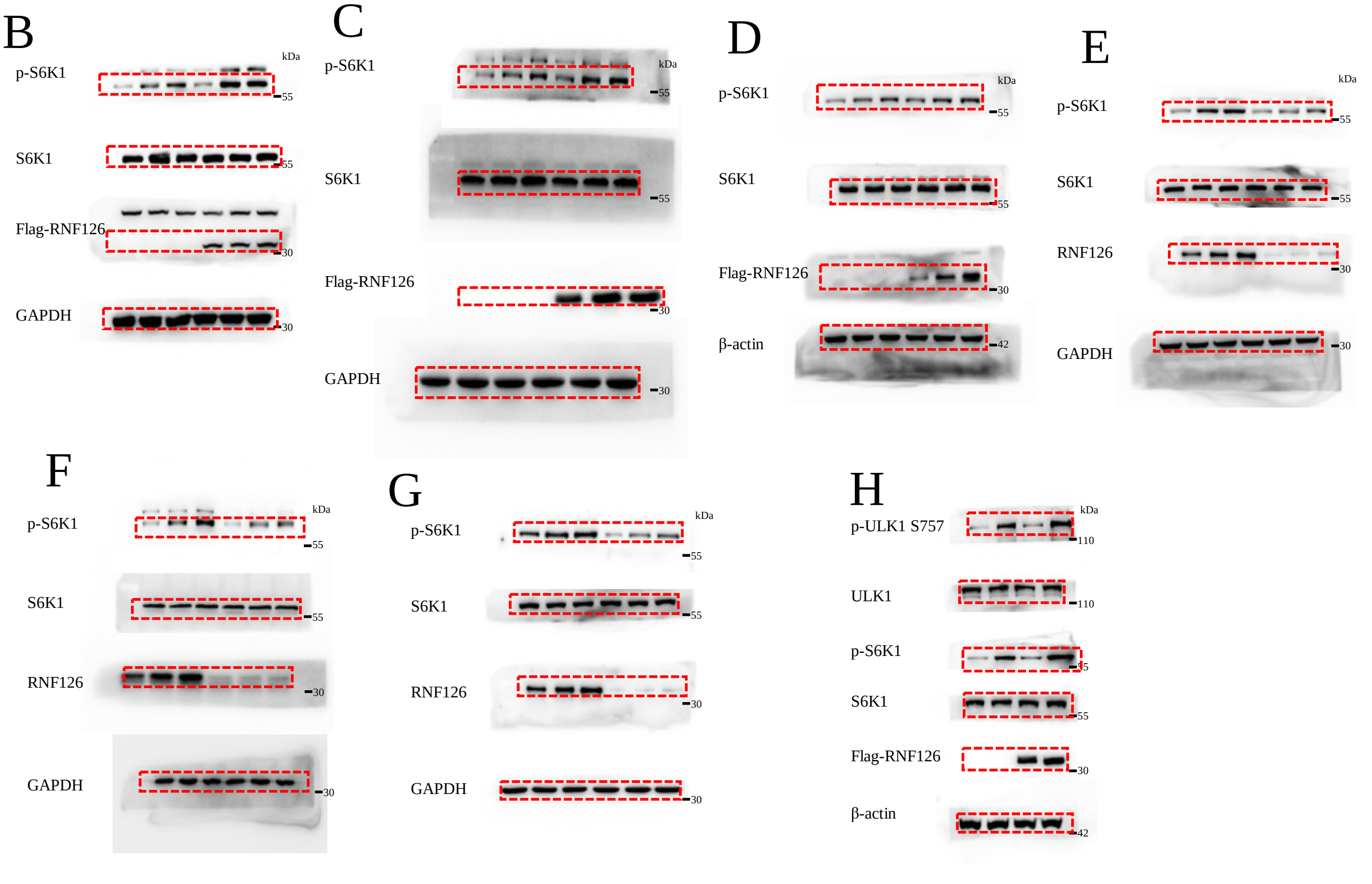
B
kDa
p-S6K1
S6K1
Flag-RNF126
GAPDH
C
kDa
p-S6K1
S6K1
Flag-RNF126
GAPDH
D
kDa
p-S6K1
S6K1
Flag-RNF126
β-actin
E
kDa
p-S6K1
S6K1
RNF126
GAPDH
F
kDa
p-S6K1
S6K1
RNF126
GAPDH
G
kDa
p-S6K1
S6K1
RNF126
GAPDH
H
kDa
p-ULK1 S757
ULK1
p-S6K1
S6K1
Flag-RNF126
β-actin

Fig. S4

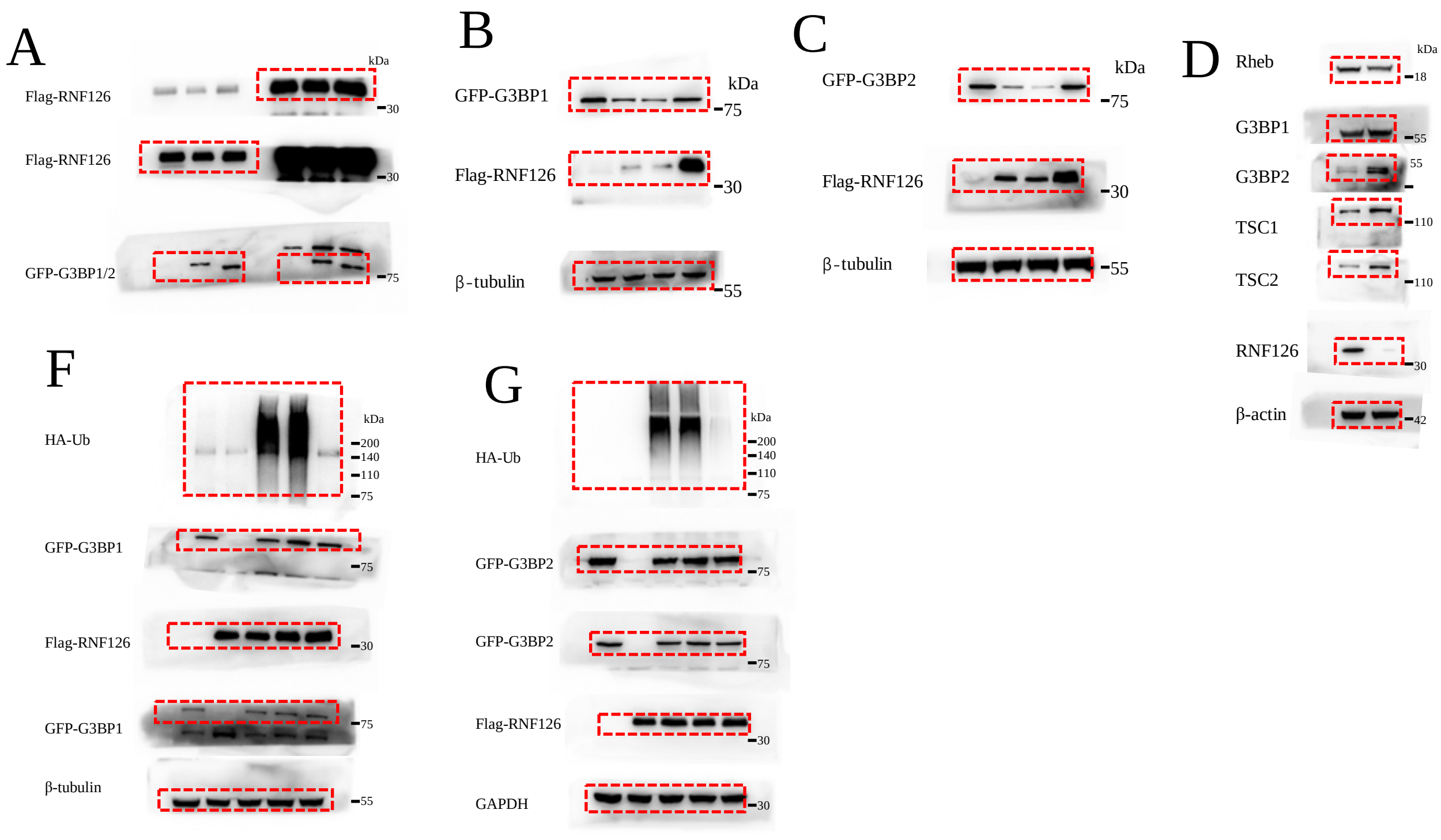
A
Flag-RNF126
Flag-RNF126
GFP-G3BP1/2
kDa
30
30
75
B
GFP-G3BP1
Flag-RNF126
β-tubulin
kDa
75
30
55
C
GFP-G3BP2
Flag-RNF126
β-tubulin
kDa
75
30
55
D
Rheb
G3BP1
G3BP2
TSC1
TSC2
RNF126
β-actin
kDa
18
55
55
110
110
30
42
F
HA-Ub
GFP-G3BP1
Flag-RNF126
GFP-G3BP1
β-tubulin
kDa
200
140
110
75
75
30
75
55
G
HA-Ub
GFP-G3BP2
GFP-G3BP2
Flag-RNF126
GAPDH
kDa
200
140
110
75
75
75
30
30

Fig. S5

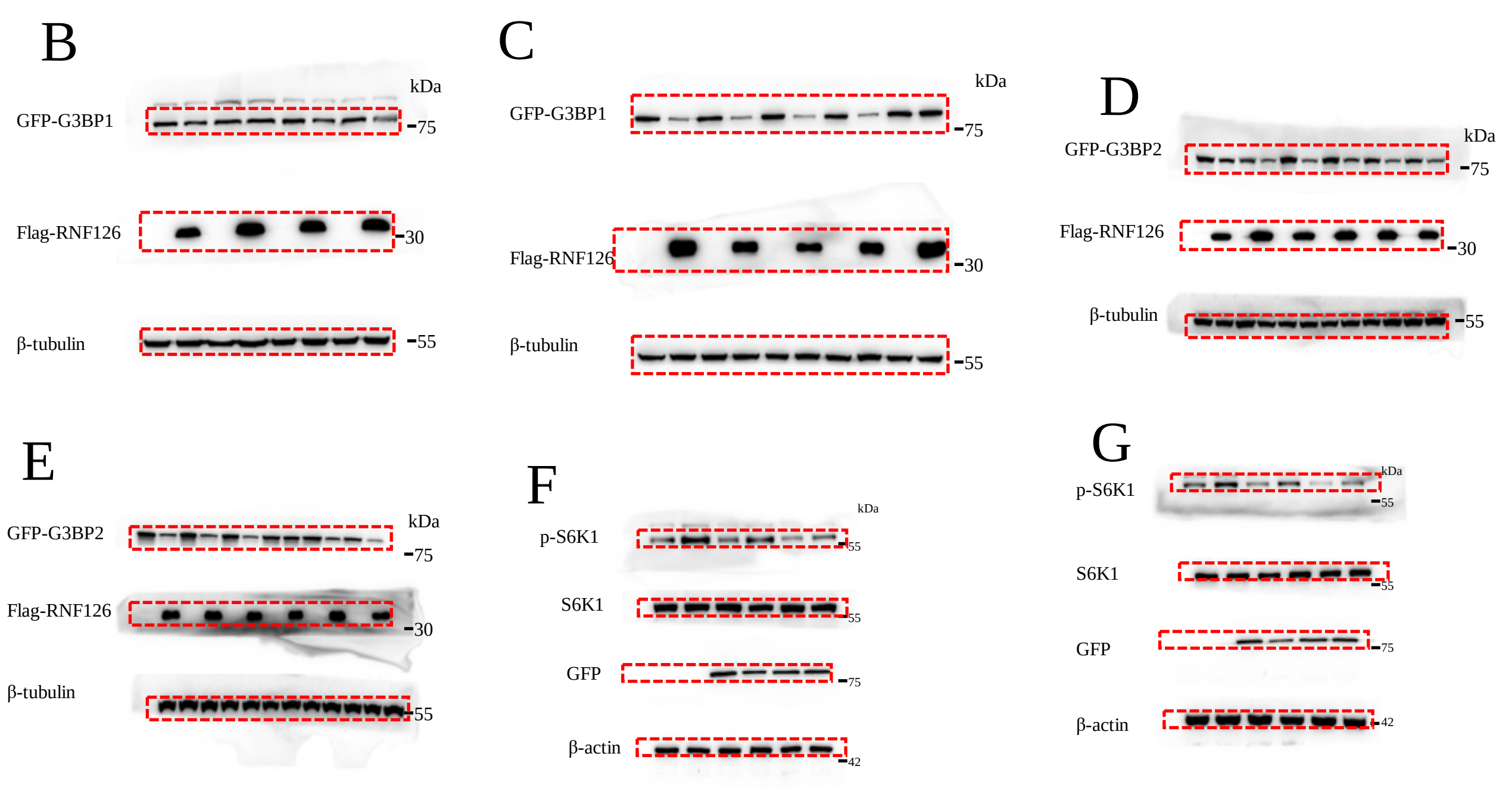
B
GFP-G3BP1
Flag-RNF126
β-tubulin
kDa
75
30
55
C
GFP-G3BP1
Flag-RNF126
β-tubulin
kDa
75
30
55
D
GFP-G3BP2
Flag-RNF126
β-tubulin
kDa
75
30
55
E
GFP-G3BP2
Flag-RNF126
β-tubulin
kDa
75
30
55
F
p-S6K1
S6K1
GFP
β-actin
kDa
55
55
75
42
G
p-S6K1
S6K1
GFP
β-actin
kDa
55
55
75
42

Fig. S6

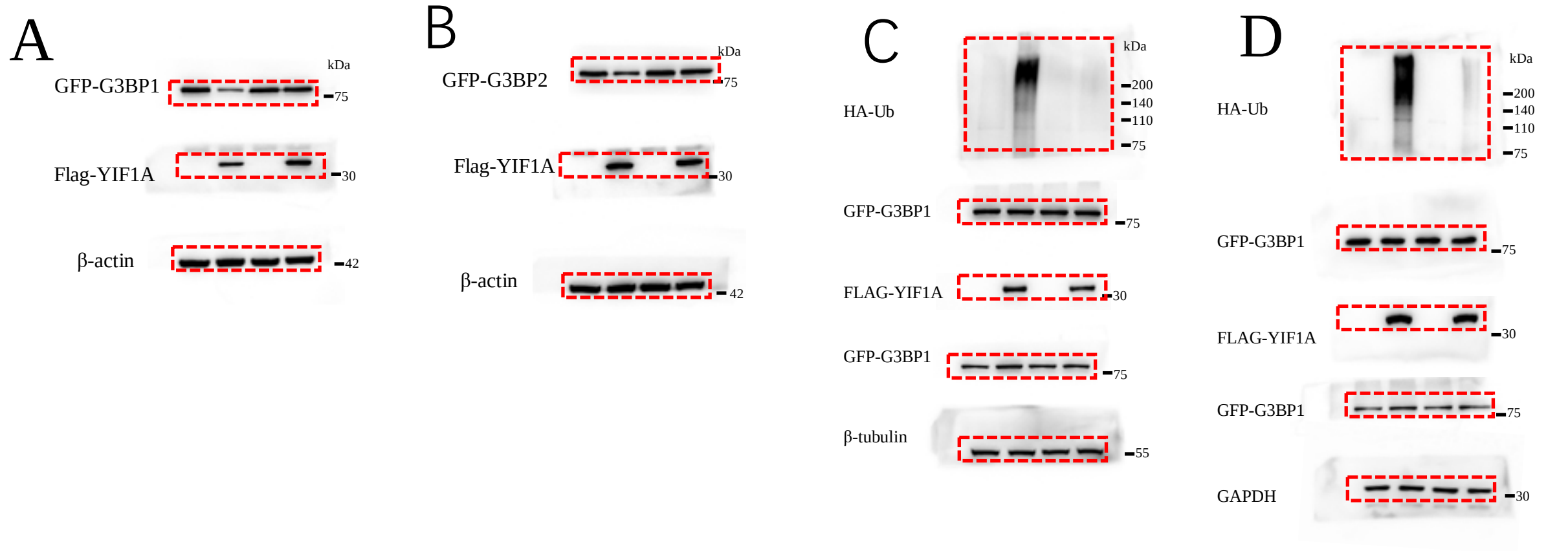
A
GFP-G3BP1
Flag-YIF1A
β-actin
kDa
75
30
42
B
GFP-G3BP2
Flag-YIF1A
β-actin
kDa
75
30
42
C
HA-Ub
GFP-G3BP1
FLAG-YIF1A
GFP-G3BP1
β-tubulin
kDa
200
140
110
75
75
30
75
55
D
HA-Ub
GFP-G3BP1
FLAG-YIF1A
GFP-G3BP1
GAPDH
kDa
200
140
110
75
75
30
75
30

Fig. S7

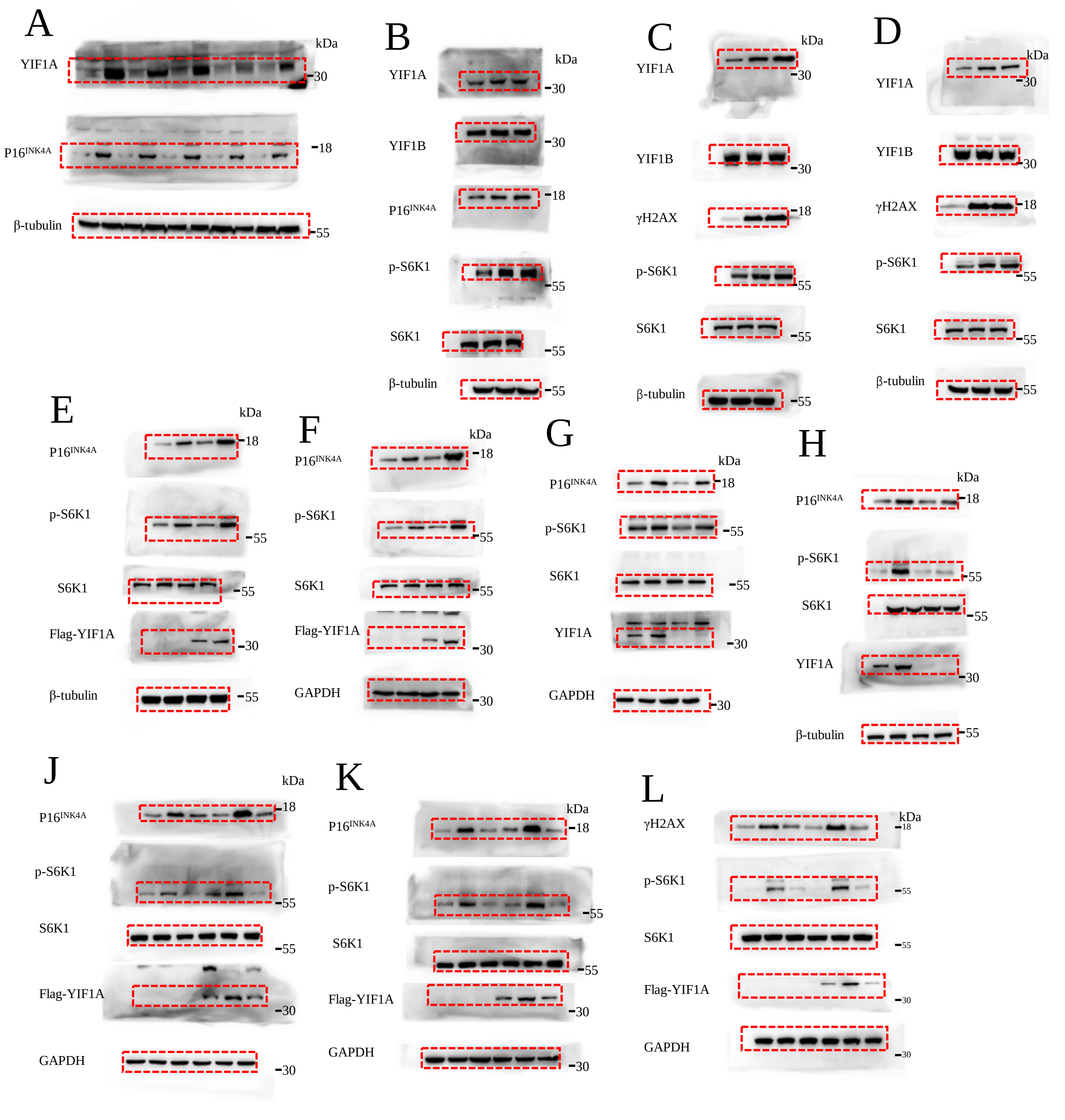
A
YIF1A
P16INK4A
β-tubulin
kDa
30
18
55
B
YIF1A
YIF1B
P16INK4A
p-S6K1
S6K1
β-tubulin
kDa
C
YIF1A
YIF1B
γH2AX
p-S6K1
S6K1
β-tubulin
D
YIF1A
YIF1B
γH2AX
p-S6K1
S6K1
β-tubulin
E
P16INK4A
p-S6K1
S6K1
Flag-YIF1A
β-tubulin
F
P16INK4A
p-S6K1
S6K1
Flag-YIF1A
GAPDH
G
P16INK4A
p-S6K1
S6K1
YIF1A
GAPDH
H
P16INK4A
p-S6K1
S6K1
YIF1A
β-tubulin
J
P16INK4A
p-S6K1
S6K1
Flag-YIF1A
GAPDH
K
P16INK4A
p-S6K1
S6K1
Flag-YIF1A
GAPDH
L
γH2AX
p-S6K1
S6K1
Flag-YIF1A
GAPDH

Fig. S8
