# Supplementary material for: YIF1A activates mTORC1 signaling to promote cellular senescence
Source: Cell Death Dis. 2026 Jun 23;17(1):778. doi: 10.1038/s41419-026-09034-z (PMC13542221; doi:10.1038/s41419-026-09034-z)
Supplement: Supplementary file 2 [file 41419_2026_9034_MOESM1_ESM.docx]

**SUPPLEMENTAL INFORMATION**

**Figure S1. YIF1A localizes at ERGIC.**

A-B, MCF7 cells were starved of serum for 50 min, or starved and re-stimulated with 100 nM insulin for 15 min. The co-localization between YIF1A and ERGIC53 (A), and Lamp1 (B) was quantified using Pearson’s correlation. Each dot is represented by an individual data. C, MCF7 cells were treated as in A. The collected cells were lysed with TRIzol reagent (Takara) and the total RNAs were then extracted to perform Real-time PCR. ns: not significant.

**Figure S2. YIF1A enhances mTORC1 signaling.**

A-D,Sum159PT cells (A), MDA-MB-231 (B), Hela (C) and NCI-H1299 (D) cells expressing empty vector (EV) or Flag-YIF1A were starved of serum for 50 min, or starved and re-stimulated with 100 nM insulin for 10 or 15 min. E-F, Sum159PT cells (E) and MDA-MB-231 (F) expressing negative control (shNC) or shYIF1A were treated as described in Figure A.G, The shYIF1A Sum159PT cells were rescued with the siRNA-resistant YIF1A plasmid for 24 h. Then, the cells were starved of serum for 50 min and re-stimulated with 100 nM insulin for 15 min. H, Sum159PT cells expressing EV or Flag-YIF1A were starved of serum for 50 min, or starved and re-stimulated with 100 nM insulin for 15 min. DMSO or 100 nM Rapamycin was pre-added for 50 min before addition of insulin. I, MCF7 cells stably expressing TMEM115-HA were starved of serum for 50 min, or starved and re-stimulated with 100 nM insulin for 15 min. The cells were then homogenized to isolate Golgi apparatus by immunoprecipitation using HA beads. J, MCF7 cells expressing EV or Flag-YIF1A were starved of serum for 50 min, or starved and re-stimulated with 100 nM insulin for 15 min. DMSO or 2 ug/ml brefeldin A (BFA) was pre-added for 8 h before addition of insulin. The data were obtained from three independent experiments and expressed as mean ± SE. ns: not significant; *P ≤0.05; **P ≤0.01; ***P ≤ 0.001; ****P < 0.0001.

**Figure S3. YIF1A promotes ubiquitin-proteasome mediated degradation of G3BP1/2.**

A, The stable shNC and shYIF1A Sum159PT cells were lysed to detect the indicated proteins. B, The stable shNC and shYIF1A MCF7 cells were lysed with TRIzol reagent and the total RNAs were then extracted to perform Real-time-PCR. C, HEK293T cells were transfected with the indicated plasmids for 24 h, and then the collected cells were lysed to perform immunoprecipitation (IP) using Flag M2 beads. D-E, HEK293T cells expressing GFP-G3BP1 (D) or GFP-G3BP2 (E) were fixed to perform immunofluorescence. F-G, The shNC and shYIF1A HEK293T cells expressing GFP-G3BP1 (F) or GFP-G3BP2 (G) were fixed to perform immunofluorescence. Each dot was represented by an individual data. H, HEK293T cells expressing the indicated proteins were incubated with or without 10 μM MG132 for 15 h prior to being lysed. I-J, HEK293T cells expressing the indicated proteins were incubated with DMSO, 1 μM bafilomycin A1 (BA) or 10 μM MG132 for 15 h prior to being lysed. K-L, HEK293T cells expressing GFP-G3BP1 (K) or GFP-G3BP2 (L) with EV or HA-ubiquitin were starved of serum for 15 h, or starved and re-stimulated with 100 nM insulin for 15 min. The collected samples were lysed to perform denaturing IP using GFP beads. 10 μM MG132 was added to cell culture medium for 15 h prior to being lysed. M-N, The shNC and shYIF1A MCF7 cells were starved of serum for 50 min, or starved and re-stimulated with 100 nM insulin for 15 min. The collected samples were lysed to perform denaturing IP using anti-G3BP1 (M) or anti-G3BP2 (N) antibodies. 10 μM MG132 was added to cell culture medium for 12 h prior to being lysed. The data were expressed as mean ± SE. ns: not significant; **P ≤ 0.01; ***P ≤ 0.001; ****P < 0.0001.

**Figure S4. RNF126 positively regulates mTORC1 signaling.**

A, HEK293T cells expressing Flag-RNF126 were starved for 15 h, or starved or re-stimulated with 100 nM insulin for 15 min. Then, cells were fixed to perform immunofluorescence. Each dot is represented by an individual data. B-D, The MCF7 (B), Sum159PT (C), and MDA-MB-231 (D) cells expressing EV or Flag-RNF126 were starved of serum for 50 min, or starved and re-stimulated with 100 nM insulin for 10 or 15 min (*n* = 3 of independent experiments). E-G, The MCF7 (E), Sum159PT (F), and MDA-MB-231 (G) cells expressing shNC or shRNF126 were treated as described in Figure B (*n* = 3 of independent experiments). H, MCF7 cells expressing EV or Flag-RNF126 were starved of serum for 50 min, or starved and re-stimulated with 100 nM insulin for 15 min (*n* = 3 of independent experiments). The data were expressed as mean ± SE. ns: not significant; *P ≤ 0.05; **P ≤ 0.01; ***P ≤ 0.001; ****P < 0.0001.

**Figure S5. RNF126 promotes the degradation of G3BP1/2.**

A, HEK293T cells expressing the indicated proteins were collected and lysed to perform IP using M2 flag beads. B-C, HEK293T cells expressing GFP-G3BP1(B) or GFP-G3BP2 (C) with or without Flag-RNF126 were incubated with 1μM bafilomycin A1 (BA) or 10 uM MG132 for 15 h. D, Sum159PT cells expressing shNC or shRNF126 were lysed to detect the indicated proteins. E, The stable shNC and shYIF1A MCF7 cells were lysed with TRIzol reagent and the total RNAs were then extracted to performe Real-time-PCR. F-G, HEK293T cells expressing GFP-G3BP1 (F) or GFP-G3BP2 (G), with or without HA-ubiquitin, HA-K48, HA-K63 and Flag-RNF126 were collected, lysed and boiled to perform denaturing IP using GFP beads. MG132 was added to the cell culture medium for 15 h prior to being lysed. The data were obtained from three independent experiments and expressed as mean ± SE. ns: not significant; *P ≤ 0.05; **P ≤ 0.01; ***P ≤ 0.001; ****P < 0.0001.

**Figure S6. The mutation of G3BP1/2 at K64 confers resistance to RNF126-mediated degradation.**

A, G3BP1/2 domain organization labeled with lysine residues on which ubiquitination has been reported. B-C, HEK293T cells expressing wide-type (WT) GFP-G3BP1 or its different mutants with or without Flag-RNF126 were lysed to blot the indicated proteins. D-E, HEK293T cells expressing WT GFP-G3BP2 or its different mutants with or without Flag-RNF126 were lysed to blot the indicated proteins. F, MCF7 cells expressing GFP-G3BP1 or GFP-G3BP1 K64R were starved of serum for 50 min, or starved and re-stimulated with 100 nM insulin for 15 min. G, MCF7 cells expressing GFP-G3BP2 or GFP-G3BP2 K64R were treated as described in Figure N. The data were obtained from three independent experiments and expressed as mean ± SE. ns: not significant; *P ≤ 0.05; **P ≤ 0.01; ***P ≤ 0.001; ****P < 0.0001.

**Figure S7. The mutation of G3BP1/2 at K64 confers resistance to YIF1A-mediated ubiquitination.**

A, HEK293T cells expressing GFP-G3BP1 or GFP-G3BP1 K64R with or without Flag-YIF1A were lysed to blot the indicated proteins. B, HEK293T cells expressing GFP-G3BP2 or GFP-G3BP2 K64R with or without Flag-YIF1A were lysed to blot the indicated proteins. C, HEK293T cells expressing GFP-G3BP1 or GFP-G3BP1 K64R with or without Flag-YIF1A and HA-ubiquitin were lysed and boiled to perform denaturing IP using GFP beads. MG132 (10 μM) was pre-added to cell culture prior to being lysed.. D, HEK293T cells expressing GFP-G3BP2 or GFP-G3BP2 K64R with or without Flag-YIF1A and HA-ubiquitin were treated as described in Figure C. The data were obtained from three independent experiments and expressed as mean ± SE. ns: not significant; **P ≤ 0.01; ****P < 0.0001.

**Figure S8.** **YIF1A enhances cellular senescence.**

A, Protein level of YIF1A in spleen, lung, liver, heart and kidney from 3-month-old (Young, Y) and 24-month-old (Aged, A) male wide-type mice. B, HEK293T cells were treated with 10 μM ETO for 24 h and then released for 2 or 3 days. C, MCF7 cells were treated with 2 μΜ or 10 μΜ ETO for 7 or 4 days, respectively. D, MCF7 cells were treated with 4 μΜ or 10 μΜ DOX for 7 or 4 days, respectively. E-F, HEK293T cells expressing EV or Flag-YIF1A were treated with 10 μΜ ETO (E) or 2 μΜ DOX (F) for 3 days. G-H, HEK293T cells expressing shNC or shYIF1A were treated with 10 μΜ ETO (G) or 2 μΜ DOX (H) for 3 days. I, MCF7 cells stably expressing shNC or shYIF1A were treated with 2 μM ETO for 7 days, and then fixed to perform immunofluorescence. J-K, HEK293T cells expressing EV or Flag-YIF1A were treated with 10 μΜ ETO (J) or 2 μΜ DOX (K) for 3 days. 100 nM rapamycin was added to culture medium following the induction of cellular senescence. L, MCF7 cells stably expressing EV or Flag-YIF1A were treated with 2 μM ETO for 7 days. 100 ng/ml brefeldin A (BFA) was added to cell culture medium following the induction of cellular senescence. M, A schematic showing the mechanism by which YIF1A regulates mTORC1 signaling pathway. The data were obtained from three independent experiments and expressed as mean ± SE. ns: not significant; *P ≤ 0.05; **P ≤ 0.01; ***P ≤ 0.001; ****P < 0.0001.
